# Protocol for a randomized controlled trial to assess the effect of Self-Management for Amputee Rehabilitation using Technology (SMART): An online self-management program for individuals with lower limb loss

**DOI:** 10.1371/journal.pone.0278418

**Published:** 2023-03-23

**Authors:** Elham Esfandiari, WC Miller, Sheena King, Michael Payne, W. Ben Mortenson, Heather Underwood, Crystal MacKay, Maureen C. Ashe

**Affiliations:** 1 Graduate Program in Rehabilitation Sciences, The University of British Columbia, Vancouver, BC, Canada; 2 GF Strong Rehabilitation Research Lab, Vancouver Coastal Research Institute, Vancouver, BC, Canada; 3 Department of Occupational Science and Occupational Therapy, The University of British Columbia, Vancouver, BC, Canada; 4 GF Strong Rehabilitation Centre, Vancouver Coastal Health, Vancouver, BC, Canada; 5 Department of Physical Medicine and Rehabilitation, Western University, London, ON, Canada; 6 International Collaboration on Repair Discoveries, Vancouver, BC, Canada; 7 West Park Healthcare Centre, Toronto, ON, Canada; 8 Department of Physical Therapy, Temerty Faculty of Medicine, University of Toronto, Toronto, ON, Canada; 9 Centre for Hip Health and Mobility, The University of British Columbia, Vancouver, BC, Canada; 10 Department of Family Practice, The University of British Columbia, Vancouver, BC, Canada; Universitat de Valencia, SPAIN

## Abstract

**Background:**

Lower limb loss (LLL) is a distressing experience with psychological, physical, and social challenges. Education is needed to enhance the coping skills and confidence of patients to improve LLL outcomes. However, access to rehabilitation services and education is limited outside of urban centers. To address this service gap, we co-created an eHealth platform, called **S**elf-**M**anagement for **A**mputee **R**ehabilitation using **T**echnology (SMART).

**Objectives:**

First, we will test the effect of SMART and usual care compared with usual care only on walking capacity and confidence among individuals with LLL. Second, we will describe key implementation factors for program delivery and adoption at the person- and provider-level.

**Methods:**

This is a Type 1 Effectiveness-Implementation Hybrid Design, mixed-methods, multi-site (British Columbia and Ontario, Canada), parallel, assessor-blinded randomized controlled trial. Participants will include adults with unilateral LLL, during early prosthetic fitting (<2 years after casting for initial prosthesis). Participants in both groups will receive usual care. The experimental group will receive SMART with weekly support sessions from a trained peer mentor for goal setting and action planning for six weeks. Participants will be encouraged to continue using SMART for an additional four weeks. The control group will receive usual care, and weekly social contacts for six weeks. The primary outcome measure is walking capacity operationalized as the performance based Timed Up and Go test. The secondary outcome is walking confidence using the Ambulatory Self-Confidence Questionnaire. Outcome measures will be assessed at baseline, immediately post-intervention, and four weeks follow-up. We will describe key implementation factors (such as, participant experience, intervention adoption, fidelity) throughout the study using questionnaires, semi-structured interviews, and direct observation.

**Results:**

No participants have been enrolled.

**Conclusions:**

SMART has the potential to provide knowledge and skill development to augment rehabilitation outcomes for adults with LLL.

**Trial registration:**

NCT04953364 in Clinical Trial Registry (https://clinicaltrials.gov/).

## 1. Introduction

### 1.1. Background

Limb loss is distressing [1] and associated with considerable psychological, physical and social challenges [2]. Each year, more than 7,300 Canadians are admitted to hospitals nationwide to undergo lower limb loss (LLL) [3]. The majority of LLLs (86%) occur in adults over 50 years of age [3]. The challenges associated with LLL include body image changes, mobility restrictions, pain [4], depression [5], social isolation, and decreased quality of life [6].

Education is an important part of rehabilitation after LLL to support and engage patients in self-management of their health condition [7–9]. Self-management programs with education and supportive interventions may increase people’s coping skills and build confidence to better manage disease-related physical and psychological challenges [7, 8]. Tailored self-management programs, using different feedback and peer support strategies, are reported to be effective at promoting positive health outcomes in other chronic conditions [10, 11]. Additionally, a community-based self-management program for individuals with LLL noted promising improvements in well-being, pain, general self-efficacy [9], and quality of life [12]. However, as rehabilitation services are a primary access-point for education [13, 14], the predominant localization of these services in Canadian urban centers limits their accessibility for some population groups [13, 14].

eHealth–the use of technologies such as computers and smart devices to support health services [15]–is considered an effective approach for delivering self-management programs [10, 15]. For example, cardiac rehabilitation delivered by eHealth may improve physical activity behavior and quality of life [16]. eHealth interventions with interactive features may also increase long-term medication adherence [16]. Further, a LLL physical activity program delivered by video calls increased weekly step count [17].

Based on evidence [18] and theory [19, 20], we co-created [21] an eHealth platform for individuals with LLL called Self-Management for Amputee Rehabilitation using Technology (SMART) [22]. SMART is a web-based app containing educational modules to guide adults with LLL to actively engage in self-management. In a pre-post mixed methods study [22, 23] with 12 individuals with LLL, we assessed the feasibility of SMART delivered with peer-support. This demonstrated SMART was an acceptable intervention, which can support individuals with LLL by providing knowledge and facilitating skill acquisition and development. Participants perceived SMART to be a complementary resource to gain knowledge and encourage them to take actions towards their self-management goals [22]. For this current study, we will conduct an assessor-blinded randomized controlled trial (RCT) and use a mixed methods approach [23] to evaluate the effectiveness of SMART on LLL-relevant outcomes compared to a control group, in older adults (≥50yrs) with unilateral LLL, while documenting implementation factors (Type 1 Effectiveness-Implementation Hybrid Design) [24].

### 1.2. Hypotheses

We hypothesize adults (50 years and older) with LLL who receive SMART and usual care (intervention group), compared with social contact calls and usual care (control group), will have greater walking capacity (Timed-Up and Go test (TUG)) after six weeks.

### 1.3. Trial Design

This is a 1:1 parallel single (assessor)-blinded RCT with a clinical superiority framework [25] using a Type 1 Effectiveness-Implementation Hybrid Design [24].

## 2. Methods

**Fig 1:**
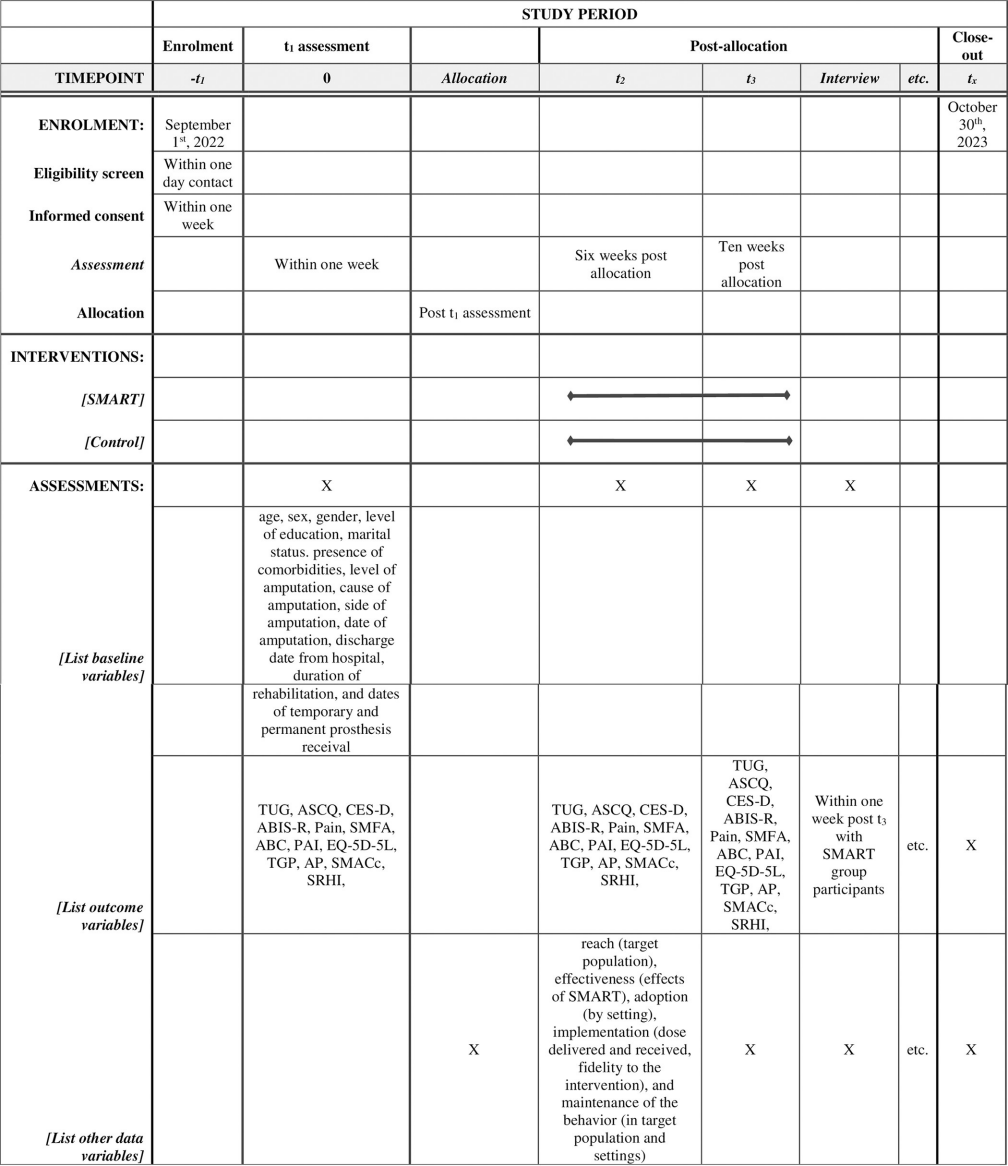
Standard Protocol Items: Recommendations for Interventional Trials (SPIRIT) schedule of enrolment, interventions, and assessments. (*TUG* = Timed Up and Go; *ASCQ* = Ambulatory Self-Confidence Questionnaire; *CES-D* = The Center for Epidemiologic Studies Depression Scale; *ABIS-R* = Revised Amputee Body Image Scale; __ *SMFA* = Short Musculoskeletal Function Assessment; *ABC* = Activities-specific Balance Confidence scale; *PAI* = Physical Activity Identity; *EQ-5D-5L* = Euro Quality of Life–Five Level Instrument; *TGP* = Tenacious goal pursuit; *AP* = Action planning scale; *SMASc* = Self-Management Assessment Scale; *SHRI* = Self-Report Habit Index).

We used the Standard Protocol Items: Recommendations for Interventional Trials (SPIRIT) to report the study protocol [26]; please see S1 Checklist. Fig 1 shows the SPIRIT schedule of enrollment, interventions, and assessments.

### 2.1. Trial setting

We will recruit from four sites, one in British Columbia and three in Ontario. In British Columbia, we will recruit potential participants with LLL from five health authorities: Vancouver Coastal Health (including Providence Health Care), Vancouver Island Health, Fraser Health, Northern Health, and Interior Health. In Ontario, we will recruit from two health regions: South West (Parkwood Institute) and Central Health (West Park Healthcare Centre, and Sunnybrook Hospital). We chose these sites for three key reasons: 1) access to a large population of individuals with LLL to enhance the recruitment; 2) the characteristics of LLL population from these sites are representative of Canadians with LLL (e.g., primarily older individuals with dysvascular-related amputation [3]) and includes some from rural and remote communities to support generalizability of findings; and 3) similarity of inpatient and outpatient rehabilitation services across the sites and provinces.

### 2.2. Participants

We will invite people who meet the following criteria to join the study: 1) aged 50 years or older; 2) have a unilateral transtibial amputation, knee disarticulation or transfemoral amputation due to diabetes or vascular disease; 3) fitted with their initial prosthesis no longer than two years; 4) self-identify as being able to speak and read English; 5) sufficient cognitive capabilities (Telephone Montreal Cognitive Assessment ≥ 19 [27]); 6) have access to the internet and a computer/smartphone. Individuals will be excluded if they have a substantial health condition (e.g., congestive heart failure, diagnosed dementia), anticipate further surgery (e.g., LLL revision), or are unable to use the computer independently (e.g., using hands for typing).

### 2.3. Trial Interventions

#### 2.3.1. Self-Management for Amputee Rehabilitation using Technology (SMART)

Participants allocated to the intervention group will receive usual care after LLL and access to SMART. We previously provided a detailed description of SMART using the Template for Intervention Description and Replication checklist [22, 28]. Participants will receive a link enabling direct connection with the SMART platform via individualized username and password. SMART link is housed on the affiliated university educational platform, and data will be kept in Canada.

SMART is a web-based eHealth intervention with six interactive, narrated modules (Table 1). There is an additional section, “More resources” which includes additional resources for LLL such as timeline after amputation, insurance, and information to locate a prosthetist. Each module is about 20 to 30 minutes in duration. SMART will be delivered with the support of peer mentors using Brief Action Planning (BAP) [29], based on motivational interviewing [30]. Six peer mentors will be recruited from either British Columbia or Ontario. Peer mentors will be more than 50 years in age and have either transfemoral and transtibial amputations. They will support participants with goal setting and action planning [31]. All peer mentors will take the online BAP training [29]. Peer mentors will provide a 20-minute weekly secure online meeting (via Zoom platform) with SMART group participants, for six weeks [29]. The peer mentors will ask the participants how long they spent on each SMART module and the adverse events in the weekly meeting. All participants in the SMART group will receive online training. The trainer will be a research assistant who will not be involved in data collection. They will arrange the first meeting between the participant and the peer mentor. Participants will be asked to complete one module each week, at their own convenience, over the 6-week intervention period. The SMART platform will be asynchronously monitored through a secure web portal by the trainer, who can observe participants’ progress and provide feedback if required. If there is no online activity in seven consecutive days, the trainer will contact the participant to inquire about the reasons and troubleshoot any problems. After six weeks (end of the intervention), the trainer will ask the participants to use SMART independently and refer back to the information for four more weeks and document their usage.

**Table 1:** Overview of Self-Management for Amputee Rehabilitation using Technology (SMART) modules.

SMART Module	Content
1. Setting-up my goals	Overviews of keys for turning intentions into behavior (e.g., goal setting, rating confidence)
2. Understanding my amputation	Levels of amputation, pain management, skin care for residual limb, taking care of diabetic foot
3. Taking care of myself	Well-being or feeling about amputation, body image, relationships, fatigue and energy conservation, diet, and weight control
4. My life at home	Home modifications for a safe environment, daily tasks after an amputation, getting up from the floor if a fall happens, mobility aids and driving
5. My prosthesis	Types and parts of a prosthesis, wearing a prosthesis, sock management, selecting a shoe for prosthesis, cleaning, and maintenance of a prosthesis
6. Getting active	Benefits of getting active and exercise, examples of physical activities, training exercises, tips for walking with a prosthesis
More resources	Additional resources for LLL such as timeline after amputation, insurance, and finding a prosthetist.

#### 2.3.2. Control Intervention

Participants randomized to the control arm will receive usual care after LLL, and a weekly social contact calls by a trainer via phone. In this call, the trainer will answer the participants’ questions regarding the study and ask about any adverse events. This weekly contact is intended to control attention bias and interpersonal interactions [32].

### 2.4. Outcome measures

#### 2.4.1. Sociodemographic and clinical characteristics

Descriptive characteristics will be collected at baseline (T1) including, age, sex, gender, level of education, and marital status. Clinical variables such as presence of comorbidities, level of amputation, cause of amputation, side of amputation, date of amputation, discharge date from hospital, duration of rehabilitation, and dates of temporary and permanent prosthesis receival will also be collected. We will ask the participants to report their visits or calls with outpatient rehabilitation services, such as a physiotherapist, physiatrist or their surgeon.

#### 2.4.2. Clinical outcome measures

We will measure the following clinical outcomes to address the two study objectives.

*Primary clinical outcome*. *Timed Up and Go test (TUG)* is a global functional measure of mobility. It includes transitions from sit to stand, initiation of gait, acceleration, deceleration and turning, and how individuals with LLL function within their environment [33]. Participants will be asked to set a standard chair (48-cm-high) and mark a 3-meter spot from the front legs of the chair. Participants will be asked to sit on the chair. When instructed, the participant will stand and walk 3 meters, turn around, walk back, and sit back in the chair [33]. The time will be reported in seconds, rounded to the nearest 0.1s. The reliability and validity for remote measuring of TUG using an iPad app have been reported for LLL population [33]. According to the prior feasibility study, conducting TUG remotely was safe [22]. We will first ensure the participants are allowed to walk without clinician supervision; otherwise, we will skip the test. A minimal detectable difference of 1.28 seconds is reported for the TUG for people with LLL [33].

*Secondary clinical outcomes*. *Ambulatory Self-Confidence Questionnaire (ASCQ)* assesses self-efficacy in walking [34]. Self-efficacy affects an individuals’ belief in performing an activity, and is a predictor for actual behavior, including walking [19, 35]. The ASCQ includes 22 items; each item is scored from zero (which indicates not at all confident) to 10 (which indicates extremely confident) [34]. A mean score will be calculated for overall ambulatory confidence, with higher scores indicating higher confidence. There is evidence for reliability and validity of the ASCQ for community-dwelling older adults [34]. It has been reported that a change more than 0.23 is indicative of change in ambulatory confidence for older adults [34].

*The Center for Epidemiologic Studies Depression Scale (CES-D)* is used to assess depressive symptoms over the past week in adults [36–38]. Depression is an important health outcome in adults with LLL. It is associated with pain and dysfunction [39, 40], and could negatively affect the use of prosthesis [41] and quality of life [42]. The CES-D includes 20 items. Each item is scored on a four-point Likert scale from zero (which indicates the least or none of the time) to 3 (which indicates the most or all of the time). Total score ranges from zero to 60, and higher scores mean higher level of distress. There is evidence for reliability [36] and validity of the CES-D for adults with LLL [36] and the general population [43]. A total score of 19 or more indicates substantial symptoms of depression [44, 45].

*Revised Amputee Body Image Scale (ABIS-R)* assesses feelings about the body experienced after amputation [37, 46]. Poorer perceived body image is associated with depression and lower level of prosthetic satisfaction [47]. The ABIS-R includes 14 items; each item is scored from zero (which indicates never happened) to two (which indicates happening all the time) [37, 46]. The overall score ranges from zero to 28; higher scores show higher body image disturbance. There is evidence for reliability and validity of ABIS-R for adults with LLL [46]. The minimal clinically important difference of 6.3 is reported for ABIS-R [48].

*Pain* is common in individuals with LLL [4, 49, 50] and can adversely affect quality of life [51]. We will assess the following: residual limb pain (pain in the remaining part of the amputated site); phantom limb pain (in missing part of the limb); and phantom sensation (in missing part of the limb) [52]. The intensity of pain and sensation in the past week will be assessed using a 10-cm visual analogue scale (VAS), ranging from zero (which indicates no pain or sensation) to 10 (which indicates the worst pain or sensation possible). The frequency of pain and sensation in the past week will be measured using the following scale: “none”, “intermittent”, “constant with variation in intensity”, and “constant with little variation in intensity”. There is evidence for validity of VAS to assess pain [53–55]. A 33% decrease in pain indicates a meaningful change [56].

*Short Musculoskeletal Function Assessment (SMFA)* assesses individuals’ perceived health status [57, 58]. The SMFA includes 46 items in three sections: difficulty with daily activities (items 1 to 25), experiencing problems because of injury (items 26 to 34), and the extent to which the person is bothered by the problems (items 35 to 46). In this study, we will use items 1 to 34, which cover the functional assessment. The last 12 items cover how patients are bothered by their symptoms. Each item is scored on a five-point Likert scale from one (which indicates not at all bothered) to five (which indicates extremely bothered). A percentage score will be calculated: higher percentage shows higher level of dysfunction. There is evidence for validity of the SMFA for individuals with lower limb vascular injury [59]. The minimal clinically important difference of 9.7 is reported for SMFA [57].

*Activities-specific Balance Confidence scale (ABC)* assesses perceived balance confidence in different ambulatory activities [60]. Fear of falling may cause restriction in participation in daily and social activities for individuals with LLL [61]; therefore, balance confidence is a determinant of quality of life [60]. The ABC includes 16 items; each item is scored from zero (which indicates not at all confident) to 100 (which indicates extremely confident). A percentage score will be calculated; higher scores mean more confident [62]. There is evidence for reliability and validity of the ABC for individuals with LLL [62]. The minimal clinically important difference of 10% is reported for ABC [63].

*Physical Activity Identity (PAI)* assesses the extent that physical activity is considered as an integral part of the concept of self [64, 65]. The PAI includes 9 items; each item is scored on a seven-point Likert scale from one (which indicates strongly disagree) to seven (which indicates strongly agree). The total score ranges from nine to 63; higher scores mean higher identity [65]. There is evidence for reliability and validity of the PAI for community dwelling adults [64].

*Euro Quality of Life–Five Level Instrument (EQ-5D-5L)* assesses five dimensions of health-related quality of life: “mobility, self-care, usual activities, pain/discomfort, and anxiety/depression” [66]. Each dimension is scored on a five-severity level: no, slight, moderate, severe, extreme problems/unable. The total score rages from zero (which indicates the worst imaginable health state) to 100 (which indicates the best imaginable health state). There is evidence for validity of EQ-5D-5L for people with LLL [67].

*Tenacious goal pursuit (TGP)* [68] assesses the tendency to persist and increase effort in pursuing goals facing obstacles. The TGP includes five items [69]. Each item is scored on a five-point Likert scale from one (which indicates “strongly agree”) to five (which indicates “strongly disagree”). The scores are reversed and summed [69]. Total score ranges from five to 25; higher scores mean higher tenacity [70]. There is evidence for validity of TGP for adults mid-to-late life [69].

*Action planning scale (AP)* assesses whether people had formed a plan which links goal-directed behavior to environmental cues by identifying when, where, and how to act [71]. AP includes five items [71]. Each item is scored on a five-point Likert scale from completely disagree to completely agree [71, 72]. The total score will be calculated by summing each item score [72]. We will assess the AP for “exercising”, “skin monitoring”, and “cleaning the prosthesis”. There is evidence for reliability of the AP for in-patients in rehabilitation facilities [71].

*Self-Management Assessment Scale (SMASc)* [73] assesses five domains that are important for an effective self-management, including “knowledge, goals for future, daily routines, emotional adjustment, and social support” [73]. SMASc includes 10 items. Each item is scored on a six-item Likert scale from one (Strongly disagree) to six (Totally agree) [73]. Each domain ranges from two to 12. Lower scores show higher needs of support for self-management. The SMASc will be slightly modified to measure self-management based upon the LLL health condition. There is evidence for reliability and validity of SMASc for people with Type II diabetes [74].

*Self-Report Habit Index (SRHI)* assesses the automaticity of a behavior and the extent to which individuals with LLL have integrated self-management tasks into their self-concept [75, 76]. We will assess the habit formation for “skin monitoring” and “cleaning the prosthesis,” which are important in managing a LLL. The SRHI includes 12 items; each item is scored on a 7-point Likert scale. The total score is calculated as mean score and ranges from one to seven; higher scores mean stronger habit [75]. There is evidence for reliability and validity of the SRHI for community dwelling adults [77].

#### 2.4.3. Implementation factors

We will follow the RE-AIM framework [74] to explore implementation factors including, reach (target population), effectiveness (effects of SMART), adoption (by setting), implementation (dose delivered and received, fidelity to the intervention), and maintenance of the behavior (in target population and settings). During the study, the research coordinator will keep detailed logs to monitor fidelity to research protocols. Also, the peer mentor will ask the participants “how long they spent on each module” (dose) and the adverse events, such as falls or pain. We will conduct and audio record 30-minute semi-structured interviews with participants in the SMART group (n = 20) to explore their experiences using SMART. The interviews will be conducted over the Zoom platform with auto transcription. A research assistant who will not be familiar with the participants will moderate the interviews. The research coordinator will call any participants who drop out of the study to record their reason(s).

### 2.5. Measuring outcomes at follow-up

**Fig 2:**
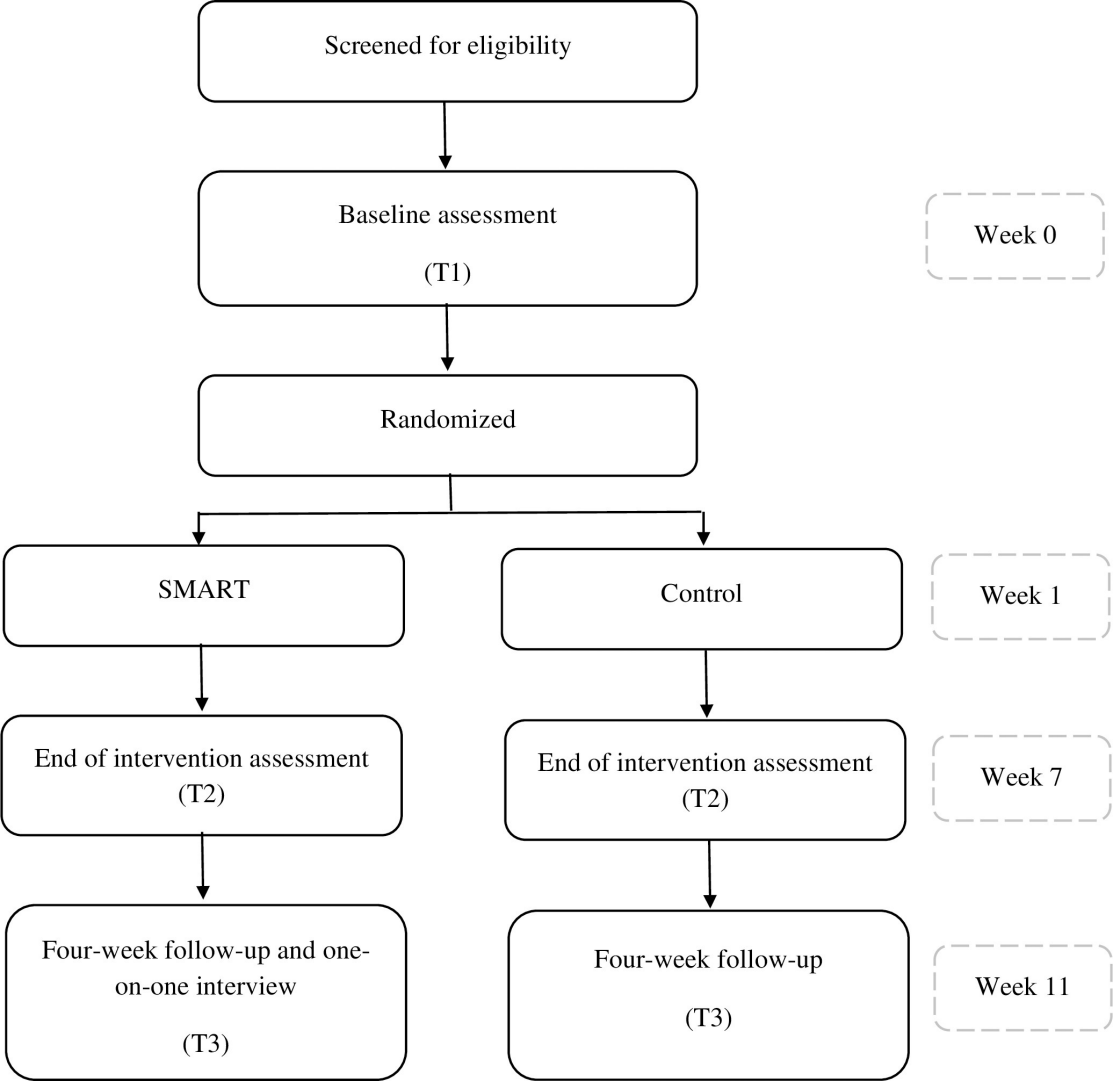
Flow diagram of study progress in terms of participants enrolment, group allocation, follow-up, and data analysis [78].

Data collection will be performed for SMART and control group at baseline (T1), within one week of completing the intervention (T2), and four weeks post-intervention (retention period) (T3). The amount and type of usual care or rehabilitation services participants received throughout the study duration, will be asked at T2 and T3. The one-on-one semi-structured interviews will be conducted at T3 with the SMART group to determine impressions of the protocol and intervention. Fig 2 shows the flow diagram of the study [78]. All data collection will occur online via Zoom utilizing a secure connection.

### 2.6. Sample Size

The sample size calculation was based on clinical superiority design [25], in which we hypothesize SMART is more effective than providing written educational information [79]. For the effectiveness portion of the study, we will aim to compare the primary outcome, TUG, using a ratio 1:1 group allocation. In our prior feasibility study, the minimal detectable difference of improvement in TUG after six weeks of using SMART was 2.1 seconds in adults with unilateral LLL (median age = 56 y) [22]. We interpreted this change as meaningful because the findings of the qualitative component of the same study revealed that participants perceived SMART and peer support could improve their mobility confidence and encourage them to take actions towards their goals, which was more walking. Assuming a minimum detectable difference of 2.1 seconds, 80% power, and type-I error probability of 0.05, we need to enroll a minimum of 38 participants per group. In our feasibility study, the retention rate was 100% [22]. However, due to challenges with participant drop out in clinical trials [80], we will account for 15% attrition rate in the sample size calculation. Therefore, we will recruit 86 participants (43 per arm). Sample size was calculated using G*Power [Version 3.1.9.4, Program written by Franz Faul, University Kiel, Germany] [81].

### 2.7. Recruitment

We will invite eligible candidates through physiotherapists, occupational therapists, prosthetists and physiatrists at the associated rehabilitation facilities, and private prosthetic clinics in British Columbia and Ontario. We will also post the study information at the hospitals and prosthetic clinics at the four sites. The electronic version of study information and a recruitment video will be posted on amputation-related web pages, such as Amputee Coalition of Canada, or social media platforms, including Facebook and Twitter. A $25 token of appreciation will be offered to all participants at the end of each evaluation timepoint. According to our feasibility study, the recruitment rate was 1.7 participants per month at British Columbia [22]; therefore, we anticipate it will take approximately 13 months to enroll 86 participants at British Columbia and Ontario sites.

### 2.8. Sequence generation and randomization

We will use a central computerized randomization process with variable block sizes to randomly allocate participants to the intervention (SMART) group or control group using a 1:1 ratio. An independent statistician will provide the randomization list through REDCap (REDCap Software, Vanderbilt University and National Institute of Health, USA). Upon the enrolment of a participant a study ID number will be allocated. The study ID number will be linked to the randomization list.

### 2.9. Allocation concealment

After completing the baseline assessment, the research coordinator will reveal the group allocation of the participant through REDCap randomization list.

### 2.10. Randomization

Interested individuals will contact the research coordinator through phone or email. The research coordinator will screen the participants and send them the consent form for their review. After expressing interest, the research coordinator will send the consent form link to participants’ emails via Qualtrics (Qualtrics Software Company, USA). Upon obtaining consent, the research coordinator will schedule the first online meeting with a blinded assessor to complete the baseline assessment. The research coordinator will log onto the online randomization system to determine the next allocation within 48 hours. The research coordinator will forward the participant’s contact information to the group trainer to schedule the first online meeting.

### 2.11. Blinding

Due to the nature of the SMART, blinding to receipt (or not) of the eHealth intervention is impossible [82]. In this study, we will hire one assessor for all data collection, who will be blinded to group allocation. We will ask participants not to disclose their group allocation during assessments [82]. Furthermore, we will have separate trainers for each group (to minimize trainer bias); and the primary outcome measure, the TUG, is a performance-based measure with standardized instructions.

### 2.12. Adherence

The SMART group trainer will monitor the activity of participants asynchronously. If there is no activity within seven consecutive days, the SMART group trainer will contact the participant. The control group will also receive a weekly contact from the trainer for six weeks. The research coordinator will contact all participants every four weeks until the end of study to remind them of the next assessment session and confirm the schedule. This strategy was found to successfully reduce the loss to follow-up to less than 20% in a previous study [83]. Furthermore, in the prior feasibility study the loss to follow-up was zero [22].

### 2.13. Data management

The research coordinator will be responsible for screening, obtaining consent, scheduling the assessment sessions, obtaining group allocation via the website, and scheduling the training session. The research coordinator will train the assessor and group trainers. The SMART group trainer will schedule the first meeting of the peer mentor and the participant. All data will be collected by a trained assessor who will be blinded to group status. A trained research assistant will be hired to complete the interviews. All quantitative and qualitative data will be password encrypted and stored in a secure server hosted by the primary affiliated university. Two research assistants will be trained to correct the Zoom auto transcription’s inaccuracies. We will also hire two research assistants to code the transcripts of interviews with the SMART group.

### 2.14. Data Analyses

We will assess the distribution of data using the one-sample Kolmogorov-Smirnov test. We will report the descriptive statistics using means and standard deviations (SD) for continuous variables and frequency and percentage for categorical variables.

#### 2.14.1. Clinical Outcomes

We will use intention-to-treat analyses [84]. All primary and secondary outcomes will be compared between the SMART and control groups, using analysis of covariance (ANCOVA), controlling for baseline scores. We will report the endpoint and change score using analysis of variance (ANOVA) to compare precision with ANCOVA for sensitivity analyses. We will conduct interim data analyses on the primary outcome when 50% of participants have completed T2. If the results show the SMART or control arm is superior, and sufficient information is available, we will end participant recruitment to minimize burden and cost. We will also disaggregate the data, such as walking capacity and confidence, by sex and gender to explore differences.

#### 2.14.2. Study and intervention fidelity, and participant experience

We will analyze transcriptions using conventional content analyses [85] with NVivo (Version 12.6.0.959); in which the coding categories will be derived directly from the transcriptions [85]. Two coders will review the data repeatedly and code them. After completing interviews, the data will be re-coded and similar codes will be grouped into themes. Themes will reflect common meanings in participants’ experiences regarding SMART. We will also use different strategies to ensure trustworthiness of the research. To support confirmability, multiple investigators will be involved in data coding. To promote dependability, the study protocol will be reported in detail [86, 87]. Reflexivity will be facilitated with self-reflection notes after each interview, involving multiple investigators in analysis, and member checking [88]. Interview results will complement the quantitative results to inform intervention fidelity, and provide a more in-depth assessment of benefits of SMART and user acceptability [23].

### 2.15. Monitoring

We will implement several strategies for monitoring. First, based on the preliminary findings of our SMART feasibility study, there was minimal risk to study participants. Only two out of 12 participants reported falls, related to LLL comorbidities such as phantom sensation at night and increasing physical activities [22]. Second, the preliminary findings showed SMART content and study protocol were acceptable [22]. SMART content also includes safety-related instruction such as avoiding extra pressure on residual limb, or tasks that can increase risk of falls. Third, at each session, the peer mentors and the control group trainer will ask the participants if they experienced any adverse events, such as falls, pain, discomfort, and report it to the research team. Fourth, all participants can contact their group trainer if they experience unusual discomfort, pain, or physical symptoms. Finally, if the trainer or the research coordinator notices any issues, they will refer the participants to their family doctor. The participants will be informed that they can discuss issues with care providers, as necessary.

Data and Safety Monitoring Board (DSMB) will review outcome data. The DSMB will provide suggestions regarding safety, SMART benefit, or study protocol modification. The DSMB will include four members who are external to the research team. The members are a biostatistician, a physiatrist, a rehabilitation therapist (occupational or physiotherapist), and an individual with LLL. The DSMB members will meet at least twice a year. Adverse events (e.g., falls, skin breakdown) will be documented by the assessor using a Treatment Protocol Checklist and will be reported to the DSMB as well as the applicable Ethics Review Board.

### 2.16. Ethics and funding

Ethics approval has been obtained from Research Ethics Boards at University of British Columbia [March 7^th^, 2022; H20-03316-A003], and regional health authorities, Sunnybrook Research Institute [April 28^th^, 2022], Joint West Park Healthcare Centre-The Salvation Army Toronto Grace Health Centre Research Ethics Board (JREB) [June 28^th^, 2022; 22-002-WP], and Western Research Ethics Board (REB). Any protocol amendments such as changes to inclusion criteria, outcomes, or analyses, will be submitted to the relevant ethics board(s) as well as the clinical trial registry. All participants will provide their informed consent electronically using an online consent form hosted on a primary affiliated university server. All data will be collected and stored anonymously using a specific participant ID number in Canada. This study has been funded by the Canadian Institutes of Health Research (CIHR) [Grant number: 438258]. We will establish data sharing, where de-identified data will be accessible upon a reasonable request after the findings are published. The results of this study will be submitted to international conferences and peer-reviewed journals for publication. We will disseminate the results electronically, including on the websites of health authorities in British Columbia and Ontario. We will provide a summary of study findings in plain language with our patient partners and share them via newsletters and websites, such as Amputee Coalition of British Columbia and Ontario Association of Amputee Care.

## 3. Results

The ethics at each university has been submitted and under review. Hiring study staff, training the peers, and group trainers at each site are currently underway. No participants have been enrolled.

## 4. Discussion

Based on the results of a previous exploratory study [22], education in rehabilitation services was limited for adults with LLL due to inaccessibility, such as living in remote areas, or due to limited time visiting the clinicians. This reduction in care creates a gap in the consolidation of education and skills to manage LLL-related outcomes which could result in decreased mobility, limited function, and quality of life [89, 90]. SMART has the potential to address this gap and improve LLL outcomes. Our study will provide an understanding about the clinical effectiveness, and key implementation factors of SMART for people with LLL. In sum, it will provide essential information for possible future integration of SMART into clinical practice to augment rehabilitation services, if it is the right fit for older adults with LLL [24].

## 5. Limitations

There are four main limitations in this study. Due to the nature of SMART, as an eHealth intervention, blinding of participants is impossible [82]. However, the outcome assessor will be blinded, and participants will be asked not to disclose their group to the assessor. Second, the participants may be receiving an ongoing rehabilitation treatment, as usual care, which could affect knowledge acquisition. Moreover, the dose and content of usual care may be different among participants. Third, while participants in SMART will be asked to complete one module per week, they may ignore the protocol. Therefore, we cannot report the precise dose of SMART as there is no tracking system for module completion. Fourth, the results may be affected by social desirability bias for self-reported outcomes.

## 6. Conclusions

This study will provide an evaluation of SMART benefits in recovery of individuals with LLL. SMART has the potential to provide accessible, low-cost educational and skill-based content regardless of geographical boundaries, which may augment current rehabilitation resources. The use of eHealth innovations for self-management and education training can promote motivation for people living with a chronic health condition to take actions to reach their goals. The eHealth innovations can be used as a complementary resource in addition to other in-person rehabilitation programs.

## Supporting information

S1 Checklist SPIRIT 2013 checklist: Recommended items to address in a clinical trial protocol and related documents*. (DOC)

S1 File SMART ethics proposal. (DOC)
