# Supplementary material for: Protocol for a randomized controlled trial to assess the effect of Self-Management for Amputee Rehabilitation using Technology (SMART): An online self-management program for individuals with lower limb loss
Source: PLoS One. 2023 Mar 23;18(3):e0278418. doi: 10.1371/journal.pone.0278418 (PMC10035895; doi:10.1371/journal.pone.0278418)
Supplement: S1 Checklist — (DOC) [file pone.0278418.s001.doc]

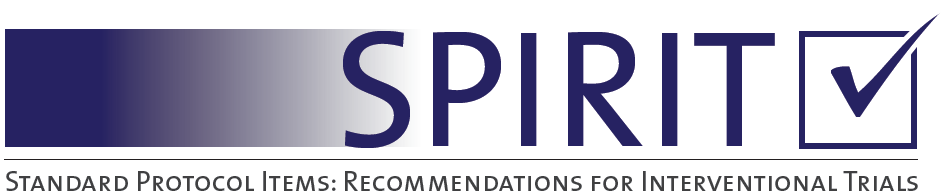


SPIRIT 2013 Checklist: Recommended items to address in a clinical trial protocol and related documents*

| Section/item | ItemNo | Description |
| --- | --- | --- |
| **Administrative information** | | |
| Title | 1 | Protocol for a Randomized Controlled Trial to Assess the Effect of Self-Management for Amputee Rehabilitation using Technology (SMART): An Online Self-Management Program for Individuals with Lower Limb Loss |
| Trial registration | 2a | NCT04953364 in Clinical Trial Registry |
|  | 2b | All items from the World Health Organization Trial Registration Data Set N/A |
| Protocol version | 3 | August 11^th^, 2022, Version I |
| Funding | 4 | Canadian Institute of Health Research (CIHR) [Grant number: 438258 for Self-Management for Amputee Rehabilitation using Technology (SMART) program: A peer supported mHealth approach for rehabilitation after lower limb amputation] |
| Roles and responsibilities | 5a | Elham Esfandiari PhD ^1, 2^: Conceptualization, investigation, methodology, project administration, writing – original draft, writing – review and editing  William C. Miller PhD, FCAOT ^1, 2, 3^: Conceptualization, investigation, methodology, project supervision, writing – review and editing  Sheena King BScPT ^4^: Conceptualization, investigation, methodology, project administration, writing – review and editing  Michael Payne MD ^5^: Investigation, methodology, project administration, writing – review and editing  W. Ben Mortenson PhD, BScOT ^1, 2, 3, 6^: Conceptualization, investigation, methodology, writing – review and editing  Heather Underwood MD ^4^: Investigation, methodology, project administration, writing – review and editing  Crystal MacKay ^7,8^: Investigation, methodology, project administration, writing – review and editing  Maureen C. Ashe PhD, BScPT ^9,10^: Conceptualization, investigation, methodology, writing – review and editing  ^1^ Graduate Program in Rehabilitation Sciences, The University of British Columbia, Vancouver, BC, Canada  ^2^ GF Strong Rehabilitation Research Lab, Vancouver Coastal Research Institute, Vancouver, BC, Canada  ^3^ Department of Occupational Science and Occupational Therapy, The University of British Columbia, Vancouver, BC, Canada  ^4^ GF Strong Rehabilitation Centre, Vancouver Coastal Health, Vancouver, BC, Canada  ^5^ Department of Physical Medicine and Rehabilitation, Western University, London, ON, Canada  ^6^ International Collaboration on Repair Discoveries, Vancouver, BC, Canada  ^7^ West Park Healthcare Centre, Toronto, ON, Canada  ^8^ Department of Physical Therapy, Temerty Faculty of Medicine, University of Toronto, Toronto, ON, Canada  ^9^ Centre for Hip Health and Mobility, The University of British Columbia, Vancouver, BC, Canada  ^10^ Department of Family Practice, The University of British Columbia, Vancouver, BC, Canada |
|  | 5b | Canadian Institute of Health Research (CIHR)  Telephone: 613-954-1968 Toll Free: 1-888-603-4178 [support-soutien@cihr-irsc.gc.ca](mailto:support-soutien@cihr-irsc.gc.ca) |
|  | 5c | Role of study sponsor and funders, if any, in study design; collection, management, analysis, and interpretation of data; writing of the report; and the decision to submit the report for publication, including whether they will have ultimate authority over any of these activities N/A |
|  | 5d | Composition, roles, and responsibilities of the coordinating centre, steering committee, endpoint adjudication committee, data management team, and other individuals or groups overseeing the trial, if applicable (see Item 21a for data monitoring committee) Page 22 |
| Introduction |  |  |
| Background and rationale | 6a | Description of research question and justification for undertaking the trial, including summary of relevant studies (published and unpublished) examining benefits and harms for each intervention Page 6 |
|  | 6b | Explanation for choice of comparators Page 6 |
| Objectives | 7 | Specific objectives or hypotheses Page 7 |
| Trial design | 8 | Description of trial design including type of trial (eg, parallel group, crossover, factorial, single group), allocation ratio, and framework (eg, superiority, equivalence, noninferiority, exploratory) Page 8 |
| Methods: Participants, interventions, and outcomes | | |
| Study setting | 9 | Description of study settings (eg, community clinic, academic hospital) and list of countries where data will be collected. Reference to where list of study sites can be obtained Page 8 |
| Eligibility criteria | 10 | Inclusion and exclusion criteria for participants. If applicable, eligibility criteria for study centres and individuals who will perform the interventions (eg, surgeons, psychotherapists) Page 9 |
| Interventions | 11a | Interventions for each group with sufficient detail to allow replication, including how and when they will be administered Page 9 |
|  | 11b | Criteria for discontinuing or modifying allocated interventions for a given trial participant (eg, drug dose change in response to harms, participant request, or improving/worsening disease) Page 21 |
|  | 11c | Strategies to improve adherence to intervention protocols, and any procedures for monitoring adherence (eg, drug tablet return, laboratory tests) Page 19 |
|  | 11d | Relevant concomitant care and interventions that are permitted or prohibited during the trial N/A |
| Outcomes | 12 | Primary, secondary, and other outcomes, including the specific measurement variable (eg, systolic blood pressure), analysis metric (eg, change from baseline, final value, time to event), method of aggregation (eg, median, proportion), and time point for each outcome. Explanation of the clinical relevance of chosen efficacy and harm outcomes is strongly recommended Page 11 |
| Participant timeline | 13 | Time schedule of enrolment, interventions (including any run-ins and washouts), assessments, and visits for participants. A schematic diagram is highly recommended (see Figure) Page 16 and Figure 1 |
| Sample size | 14 | Estimated number of participants needed to achieve study objectives and how it was determined, including clinical and statistical assumptions supporting any sample size calculations Page 17 |
| Recruitment | 15 | Strategies for achieving adequate participant enrolment to reach target sample size Page 17 |
| **Methods: Assignment of interventions (for controlled trials)** | | |
| Allocation: |  |  |
| Sequence generation | 16a | Method of generating the allocation sequence (eg, computer-generated random numbers), and list of any factors for stratification. To reduce predictability of a random sequence, details of any planned restriction (eg, blocking) should be provided in a separate document that is unavailable to those who enrol participants or assign interventions Page 18 |
| Allocation concealment mechanism | 16b | Mechanism of implementing the allocation sequence (eg, central telephone; sequentially numbered, opaque, sealed envelopes), describing any steps to conceal the sequence until interventions are assigned Page 18 |
| Implementation | 16c | Who will generate the allocation sequence, who will enrol participants, and who will assign participants to interventions Page 18 |
| Blinding (masking) | 17a | Who will be blinded after assignment to interventions (eg, trial participants, care providers, outcome assessors, data analysts), and how Page 19 |
|  | 17b | If blinded, circumstances under which unblinding is permissible, and procedure for revealing a participant’s allocated intervention during the trial Page 19 |
| **Methods: Data collection, management, and analysis** | | |
| Data collection methods | 18a | Plans for assessment and collection of outcome, baseline, and other trial data, including any related processes to promote data quality (eg, duplicate measurements, training of assessors) and a description of study instruments (eg, questionnaires, laboratory tests) along with their reliability and validity, if known. Reference to where data collection forms can be found, if not in the protocol Page 11 |
|  | 18b | Plans to promote participant retention and complete follow-up, including list of any outcome data to be collected for participants who discontinue or deviate from intervention protocols Page 19 |
| Data management | 19 | Plans for data entry, coding, security, and storage, including any related processes to promote data quality (eg, double data entry; range checks for data values). Reference to where details of data management procedures can be found, if not in the protocol Page 20 |
| Statistical methods | 20a | Statistical methods for analysing primary and secondary outcomes. Reference to where other details of the statistical analysis plan can be found, if not in the protocol Page 20 |
|  | 20b | Methods for any additional analyses (eg, subgroup and adjusted analyses) Page 21 |
|  | 20c | Definition of analysis population relating to protocol non-adherence (eg, as randomised analysis), and any statistical methods to handle missing data (eg, multiple imputation) Page 20 |
| **Methods: Monitoring** | | |
| Data monitoring | 21a | Composition of data monitoring committee (DMC); summary of its role and reporting structure; statement of whether it is independent from the sponsor and competing interests; and reference to where further details about its charter can be found, if not in the protocol. Alternatively, an explanation of why a DMC is not needed Page 22 |
|  | 21b | Description of any interim analyses and stopping guidelines, including who will have access to these interim results and make the final decision to terminate the trial Page 20 |
| Harms | 22 | Plans for collecting, assessing, reporting, and managing solicited and spontaneously reported adverse events and other unintended effects of trial interventions or trial conduct Page 21 |
| Auditing | 23 | Frequency and procedures for auditing trial conduct, if any, and whether the process will be independent from investigators and the sponsor N/A |
| Ethics and dissemination | | |
| Research ethics approval | 24 | Plans for seeking research ethics committee/institutional review board (REC/IRB) approval Page 22 |
| Protocol amendments | 25 | Plans for communicating important protocol modifications (eg, changes to eligibility criteria, outcomes, analyses) to relevant parties (eg, investigators, REC/IRBs, trial participants, trial registries, journals, regulators) N/A |
| Consent or assent | 26a | Who will obtain informed consent or assent from potential trial participants or authorised surrogates, and how (see Item 32) Page 18 and 22 |
|  | 26b | Additional consent provisions for collection and use of participant data and biological specimens in ancillary studies, if applicable N/A |
| Confidentiality | 27 | How personal information about potential and enrolled participants will be collected, shared, and maintained in order to protect confidentiality before, during, and after the trial Page 20 |
| Declaration of interests | 28 | Financial and other competing interests for principal investigators for the overall trial and each study site Page 22 |
| Access to data | 29 | Statement of who will have access to the final trial dataset, and disclosure of contractual agreements that limit such access for investigators Page 23 |
| Ancillary and post-trial care | 30 | Provisions, if any, for ancillary and post-trial care, and for compensation to those who suffer harm from trial participation N/A |
| Dissemination policy | 31a | Plans for investigators and sponsor to communicate trial results to participants, healthcare professionals, the public, and other relevant groups (eg, via publication, reporting in results databases, or other data sharing arrangements), including any publication restrictions Page 23 |
|  | 31b | Authorship eligibility guidelines and any intended use of professional writers N/A |
|  | 31c | Plans, if any, for granting public access to the full protocol, participant-level dataset, and statistical code N/A |
| Appendices |  |  |
| Informed consent materials | 32 | Model consent form and other related documentation given to participants and authorised surrogates Document in appendix |
| Biological specimens | 33 | Plans for collection, laboratory evaluation, and storage of biological specimens for genetic or molecular analysis in the current trial and for future use in ancillary studies, if applicable N/A |

*It is strongly recommended that this checklist be read in conjunction with the SPIRIT 2013 Explanation & Elaboration for important clarification on the items. Amendments to the protocol should be tracked and dated. The SPIRIT checklist is copyrighted by the SPIRIT Group under the Creative Commons “[Attribution-NonCommercial-NoDerivs 3.0 Unported](http://www.creativecommons.org/licenses/by-nc-nd/3.0/)” license.
