# Supplementary material for: Protocol for a randomized controlled trial to assess the effect of Self-Management for Amputee Rehabilitation using Technology (SMART): An online self-management program for individuals with lower limb loss
Source: PLoS One. 2023 Mar 23;18(3):e0278418. doi: 10.1371/journal.pone.0278418 (PMC10035895; doi:10.1371/journal.pone.0278418)

**Visual Abstract**

Using a participatory action research approach,^1^ we co-created the Self-Management for Amputee Rehabilitation using Technology (SMART) web-based app to guide adults with Lower Limb Amputation (LLA) to actively engage in self-management. In the current proposed study, we will use a Type 1 Effectiveness-Implementation Hybrid Design^2^ randomized controlled trial (RCT) to evaluate the 6-week effectiveness of SMART on patient-relevant outcomes, while documenting implementation factors.

**
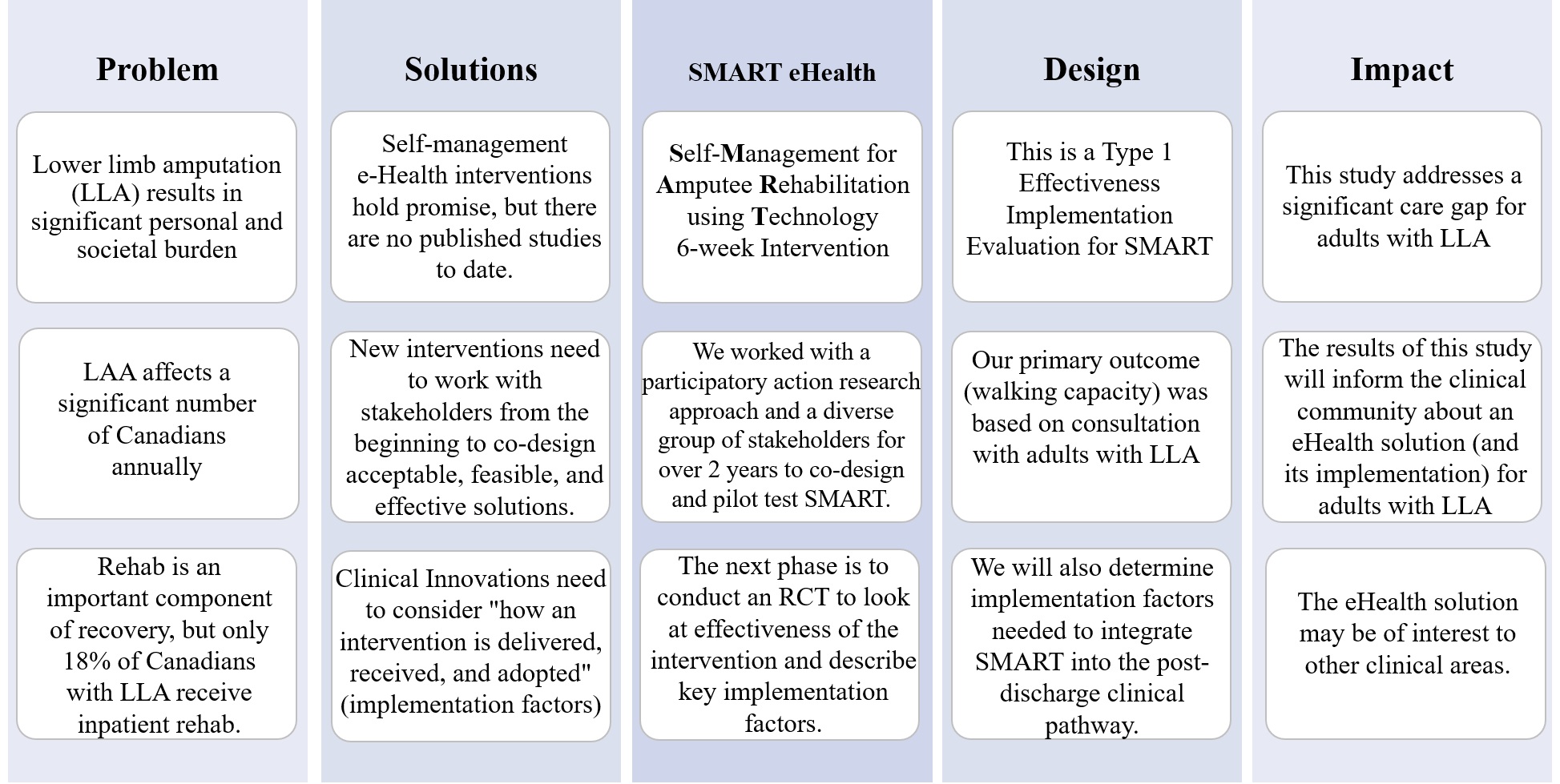
**

Figure 1: Visual Abstract for SMART Proposal

**1. THE NEED FOR A TRIAL**

**1.1 Problem:** Amputation of the lower limb (LLA) can be devastating. At the individual level it reduces physical, emotional, and social wellbeing; while contributing to a significant burden on the healthcare system. In 2017, Imam and Nominated Principal Applicant (NPA) **Miller** et al., reported that 7,300 Canadians have a LLA each year-- mostly (85%) occurring in >50 years of age.^3^ The primary cause of LLA was related to diabetes and vascular complications (91%),^3^ and most (~70%) LLA occurred in men.^3^ For many patients, undergoing an LLA predicts poor health outcomes.^4^ Women are more likely to be readmitted to hospital compared to men, due to complications after LLA,^4^ because of greater risk of morbidity and mortality after LLA including a higher risk for falls.^5-7^ Women also experience more psychological and social challenges,^8-10^ including a lack of confidence,^7, 11^ fear of falling,^7, 12^ depression^7, 13^ and pain.^8^ Combined or alone, these factors create critical barriers to independence in daily tasks (e.g., toileting) and engagement in social activities (e.g., meeting friends, volunteering).^7^ Taken together, LLA is a significant life event, that requires a wholistic approach during recovery to address both physical and psychological barriers post LLA to maximize a person’s autonomy and quality of life. Additionally, the COVID-19 pandemic has impacted the delivery and accessibility of healthcare services.

Canadian cost estimates of LLA are not available, but the projected lifetime cost of an LLA per person was estimated (in 2002) at US $509,000.^14^ The cumulative

national hospital costs associated with amputation was estimated to be more than $8.3 billion at US in 2009.^15^ In addition, the total cost for care for diabetes related LLA for 3,400 patients was exceeded $206 million in 2012 US.^16^ In the United Kingdom, the economic burden associated with diabetes-related amputations was £43.8 million for inpatient care.^17^ The cost for post amputation care (i.e., prosthetic care, physical therapy, transport, wheelchair use) was £20.8 million at United Kingdom.^17^ In Canada, there is more reliance on outpatient rehab due to high inpatient costs: 37 days is the average length of stay for an inpatient program.^18^ Despite evidence inpatient rehab is superior to outpatient,^19^ Imam and **NPA** **Miller** et al., found that only 18% of adults with LLA received inpatient rehab in Canada,^18^ and rehab for majority of patients was reliant on outpatient services. This is especially concerning as rehab services are more available in urban than remote regions.^20, 21^ As LLA affects a significant number of Canadians, and given the current trend for rehab services to be provided outside of the inpatient setting, coupled with the COVID-19 pandemic, new delivery strategies are urgently needed.

Self-management is defined as the provision of education and supportive interventions to increase patients’ coping skills and build confidence to better manage disease-related physical and psychological challenges.^22, 23^ Delivery of self-management programs through home computers, smart phones or tablets (eHealth),^24^ is emerging as an important option to deliver and manage health conditions in the community-setting, particularly during COVID-19. In general, tailored self-management programs, using feedback and peer support,^25, 26^ are effective interventions in other chronic conditions (such as diabetes). These interventions

also show effectiveness when delivered via eHealth.^25^ Specifically after LLA, education and skill development can support the recovery process, especially for older adults.^27^ However, to date, based on our review of the published evidence, there are only five publications^28-32^ using telehealth with people after LLA, and only one face-to-face study^33^ which tested effectiveness of self-management interventions after LLA. That is, we could not identify any published eHealth interventions using self-management for adults with LLA. Given the number of Canadians with a LLA each year, the declining emphasis on inpatient rehab, it is imperative to work with key stakeholders to develop an test acceptable and feasible rehab options. eHealth solutions post LLA have the potential to increase access and improve health outcomes,^27, 33^ but we need Canadian data before this delivery mode can be integrated into clinical practice. We completed foundation work with stakeholders (e.g., adults with LLA, clinicians, community organizations, etc.) to develop and test feasibility for an eHealth intervention^34-36^ called **S**elf-**M**anagement for **A**mputees using **R**ehabilitation **T**echnology (SMART) that is ready to test effectiveness, and describe key implementation factors for future integration into clinical practice, if appropriate.

**1.2. Principal research questions:** We will use a Type 1 Effectiveness-Implementation Hybrid Design^2^ randomized controlled trial (RCT) to evaluate the 6-week effectiveness (and 4-week retention effect) of SMART on patient-relevant outcomes, while documenting implementation factors. Please note, we chose the primary (and secondary) outcomes based on feedback from adults with LLA who identified walking (e.g., mobility) and new identity as of great importance in the recovery process. Our research questions are:

**Q_1_.** What is the effect of SMART on walking confidence and capacity in adults aged over 50 years with a LLA?

**Q_2_.** What is the effect of SMART on physical activity identity, knowledge, self-efficacy, and falls confidence in adults aged over 50 years with a LLA?

**Q_3_.** What are the essential factors required to implement SMART into clinical practice?

We hypothesize that older adults with LLA who receive usual care and the SMART intervention, compared with usual care, paper-based education, and social visits (control group intervention) will have greater positive change in:

**H_1_.** walking capacity, operationalized as higher change score with the two-minute walk test;^37^

**H_2_.** physical activity identity, knowledge, self-efficacy, and falls confidence, operationalized as higher change score with the physical activity identity questionnaire,^38^ patient activation measure,^39^ and activities specific balance confidence scale.^40^

**Work completed to support this proposal:** We have completed a number of key feasibility and development studies to support this next phase of testing effectiveness of the innovation (SMART eHealth intervention) and describing its implementation. Using a participatory action research approach,^1^ we created a Steering Group with our clinical co-investigators (Co-applicants (Co-A) clinicians **King, Underwood, Pousett and Moe** and patient partners **Ziehr, Doherty, Asomoza)**. We met 8 times over two years. We co-created the SMART web-based app (please see **Appendix A**), designed to guide adults with LLA to actively engage in self-management. The app is asynchronously monitored through a website portal. There were 31 clinicians, and 26 adults with LLA (73% men) who engaged in semi-structured interviews to identify common themes in the recovery process, and app content to best augment existing LLA rehab. Key findings were: adults with LLA (i) requested tools and strategies to return to walking and independence in activities of daily living (ADLs); and (ii) perceived a new identity after amputation. This feedback was incorporated into the SMART intervention, for required content and the need for peer support to enact the principles of self-management.^34, 36^ In parallel, we conducted a systematic review and meta-analysis of telehealth interventions for patients with lower limb musculoskeletal surgery^41^ to identify effective behavior change techniques (BCTs)^42, 43^ to incorporate into SMART. Self-efficacy^44^ and action planning theory^45^ informed the development of SMART. We were also guided by the Theoretical Domains Framework^46, 47^ to incorporate relevant BCTs within SMART. For example, social support, instruction on how to perform the behavior, self-monitoring behavior, etc. We currently completing a pilot study with a mixed method approach to address design and SMART intervention fidelity with 10 participants who have lived with a LLA for at least 1 year.^48^

**1.3 Why the trial is needed now:** Self-management programs reduce the number and length of hospitalizations in a variety of chronic disease conditions;^49^ subsequently reducing health risks, restoring function and independence, and promoting quality of life.^49^ On-line health education is lauded for being interactive and enabling older adult learners to re-engage over sustained periods, at their own convenience,^50^ while improving quality of care^51^ and facilitating adherence to treatment.^24^ eHealth enhances communication between patients and health care providers,^52^ and reduces costs while increasing access to health care and evidence-based health information. eHealth initiatives also offer an avenue for continued service delivery during the COVID-19 pandemic. However, to date, only one self-management program (published in 2009) was developed and evaluated for adults with LLA.^33^ The program, while promising, had a number of important limitations including: i) no involvement of trained peer facilitators [involving peers (e.g., social support) which is critical in the success of self-management programs^53^]; ii) missing knowledge content such as prosthetic and residual limb management, physical activity, and weight control: all identified as critical factors in our exploratory study^34, 36^ and in the literature^54^; and iii) face-to-face program delivery, which limits accessibility to programs to people who live in close proximity or are mobile enough to attend. Therefore, online approaches may offer greater access to self-management for people with LLA. We co-designed SMART an eHealth intervention to augment current rehabilitation and improve outcomes for people with LLA. The time is right now to conduct this trial given (i) our substantial ground work in this area; (ii) the number of people with LLA; (iii) the reduction in available inpatient rehab options; (iv) the widespread access of the internet by adults and older adults (in BC, 86% of adults 45-64 years and 55% >65 years have access to internet at home use^55^), and (v) a significant healthcare cost associated with LLA (*Section 1.1*).^14-17^

**1.4 How the results will be used:** Currently adults with LLA receive fewer hours of rehab than is considered best practice, and limited management post-discharge.^18^ This reduction in care creates a gap in the consolidation of skills and understanding, potentially leading to decreased mobility, functional independence, quality of life,^19, 56^ and a significant healthcare cost.^14-17^ SMART has the potential to address the gap and improve LLA outcomes. Our study will enable us to assess the clinical effectiveness, and key implementation factors, of SMART with a sample of people with LLA, with attention given to sex, gender and cause of amputation. In sum, it will provide essential information for integration of SMART into clinical practice, if appropriate.^2^ This study will provide us with a better understanding of the effects of SMART on walking capacity and relevant outcomes, while observing and gathering information on implementation of SMART in two provinces (BC and ON).

Beyond the potential for scale up and spread of SMART as a clinical innovation, we anticipate the results will be of interest to clinicians locally and nationally. As eHealth is relatively new as an intervention in clinical practice beyond post-amputation, we anticipate our methods will be of interest to other clinical areas. We also anticipate the findings from this study will provide valuable information on service provision via eHealth during the COVID-19 pandemic. Therefore, for our end-of-grant KT strategies we will disseminate findings via websites (e.g., health authorities in BC and ON), electronic and print newsletters (e.g., Physiotherapy Association of BC and ON), blogs, present at provincial practice forums, national webinars, and through social media (e.g., Twitter). We will build on our existing partnerships to reach members of relevant associations, such as the International Society of Prosthetics and Orthotics Canada, and provide summaries for their websites and electronic newsletters. We will submit abstracts to international conferences (e.g., International Society of Prosthetics and Orthotics World Congress), and manuscripts will be submitted for publication. We will work with our patient partners to share plain language summaries with other adults with LLA via newsletters, social media and websites (e.g. Amputee Coalition of BC).

**1.5 Possible risks associated with trial:** Based on our systematic review of telehealth interventions for people with LLA, the risk is minimum. In addition, based on our preliminary findings of the pilot work, risks associated with SMART is minimum. The preliminary results showed the promising quality of content as well as the acceptability of study protocol and assessments. The SMART content incorporates extensive safety-related material, including the identification of risks and avoiding unsafe situations (e.g., identification of potential skin breakdown on the residual limb, falls). Also, all participants can contact the group trainer (SMART or control group trainer) or the study coordinators immediately if they experience unusual discomfort, pain or physical symptoms. Additionally, participants can discuss the issues with care providers. Conducting this study is critical to explore issues, such as over exertion or residual limb skin breakdown due to promotion of physical activity.^57^ It is also possible that participants could experience altered emotional status from not meeting goals.^58^

# 2. the proposed trial

**2.1 Study Design:** This is a Type 1 Effectiveness-Implementation Hybrid Design^2^ 1:1 parallel single (assessor)-blinded randomized controlled trial (RCT) conducted online with participants from four Canadian sites (B.C.; London, ON; and two in Toronto, ON) to evaluate the 6-week effectiveness (and the 4-week retention effect) of SMART on patient-relevant outcomes, while documenting implementation factors.^59^ Following the [RE-AIM](http://www.re-aim.org/) framework, a planning tool for implementation evaluation,^60^ we will assess the implementation of SMART. Also, a small end-of-trial qualitative study will be used to understand participants’ experience.^61^

**2.2 Trial Interventions:** Participants allocated to the intervention group will receive usual care after the LLA and access to **SMART**. We provide a detailed description of SMART using the TIDieR (Template for Intervention description and Replication) checklist^62^ provided in **Appendix B**. In **Appendix C** we provide a list of BCTs adopted for each module according to our systematic review and meta-analysis.^41^ Participants will access the SMART platform using their own computer/tablet, and will be screened for having a computer/tablet before enrolling in the study. In rare cases where a participant in B.C. does have a tablet, they may receive a tablet (Apple’s iPad) preloaded with a dedicated icon to enable direct connection with the SMART platform, if needed. Tablets will not be able to be provided to participants in Ontario.

**Content:** SMART is web-based eHealth intervention that has six educational and skill development modules. SMART is accessible through this link: <https://360.articulate.com/review/content/1afbca12-f6bb-465e-b40f-1029a6fa981b/review>

We will ask participants to complete one module each week, at their own convenience, over the 6-week intervention period. Module 1 overviews the SMART program as well as ‘self-regulatory skills,’ which are key for turning intentions into behavior (e.g., goal setting, rating confidence). Other modules include managing mental health wellness (managing depression and distress, body image and sexual health), physical health (skin of the sound limb, diet and weight control, pain management, falls prevention and management), residual limb management (skin care, positioning and shaping of residual limb), managing a prosthesis (wearing a liner, sock management), and managing daily living activities (transferring to bath tub, driving). The modules are 20-30 minutes in duration and consist of goal setting, instructional videos of peers, and narrative power point presentations. Each module starts with an overview, includes evaluation of the previous module’s goals, and ends with goal setting. There is also a resources icon which provides more information and resources based on the questions and issues most-frequently raised in our focus groups, such as journey and timeline after LLA, and funding support for prostheses.

**Delivery:** SMART will be delivered with the support of peers using Brief Action Planning (BAP) tool,^63^ based on motivational interviewing^64^ which assists with goals setting and action planning and can enhance self-efficacy with self-management. Six peer facilitators identified by our clinician partners will be recruited in BC and ON. Peers are mentors with LLA who support, act as role models,^65^ and facilitate motivation using/establishing goal setting and action planning. Peers will take the online BAP training ([Centre for Collaboration, Motivation and Innovation](https://ccmi.learnupon.com/store/300131-brief-action-planning-certification)) [**Appendix D**].^63^ Peer facilitators will be assigned to each study site (BC and ON), and will provide a 20-minute weekly telephone call or Zoom video call, hosted by the University of British Columbia, to the SMART group participants, for 6 weeks.^63^ This call will help the participant set their goals, promote BAP, evaluate weekly progress, and provide support. All participants in the SMART group will receive online training on the online SMART platform from the trainer, who will be a researcher with clinical background with more than 10 years clinical and research experience with people with LLL. In British Columbia, participants will also be recruited from sites in the different health authorities: Fraser Health (Abbotsford Regional Hospital and Cancer Centre, Chilliwack General Hospital, Peace Arch Hospital, Ridge Meadows Hospital, Surrey Memorial Hospital, and Queens Park Health Care Centre), Interior Health (Kelowna General Hospital), Northern Health (Prince George Hospital), and Vancouver Island Health Authority (Victoria General Hospital, and Nanaimo Regional General Hospital). The trainer will arrange the first online meeting with the peer facilitator. The SMART platform will be asynchronously monitored through a web portal by the trainer, who observes participant progress and provides feedback if required. If there is no online activity within a 7-day (consecutive) period, the trainer will contact the participant to inquire about the reasons for inactivity and troubleshoot any problems.

**Control Intervention:** Participants in the control arm will receive usual care after LLA, education, and social calls. The education booklet is typically offered to patients at discharge, and includes information about LLA consequences, adjusting to amputation, pain management, limb positioning and exercises, mobility (e.g., wheelchair safety, hopping, and transfers), residual limb management (e.g., skin care and diabetic foot care), tips for preparing the home, and driving. A single weekly structured email or telephone contact (based on preferences, n=6 in total) will be made by the trainer. The purpose of the contact is to answer any questions, and to help control for attention bias.^45^

**2.3 Allocation - Sequence Generation and Randomization:** We will use a central computerized randomization process with variable block sizes to randomly allocate the participants to the intervention (SMART) group or control group using a 1:1 ratio by an independent statistician.

An independent statistician will provide the randomization list through REDCap (REDCap Software, Vanderbilt University and National Institute of Health, USA). The research coordinator will obtain written informed consent (Over Qualtrics platform) and a blinded assessor will complete the baseline assessment. The research coordinator will meet with the participant over Zoom (a secure, UBC-based platform) to guide them through the consent form and answer any questions. At the completion of baseline assessment, the research coordinator will log onto the system to determine the next allocation. The research coordinator will work with the group trainers to set up an appointment. Our protocol for all participants is to complete the baseline assessment, randomization and training within two weeks.

**2.4 Protecting against bias - Blinding:** Blinding of participants for the RCTs on the effect of eHealth interventions is impossible because of nature of intervetnions.^66^ Therefore, it is difficult to blind participants to the intervention received. The assessors will be blinded to group allocation, we will complete the primary outcome (TUG and ASCQ) first, and participants will be asked not to disclose their group allocation during assessments.^66^ Furthermore, we will have separate trainers for each group (to minimize trainer bias); and the primary outcome measure, the TUG is a performance-based measure with standardized instructions. Finally, all statistical analyses will be conducted by a blinded statistician.

**2.5 Inclusion and Exclusion Criteria:** To be included, participants will: have a unilateral dysvascular- or diabetic- related LLA (transtibial or transfemoral);^3^have conducted the casting for their initial prosthetic limb (up to 2 years); be aged ≥ 50 years (85% of adults with LLA);^3^ have a computer or tablet and have access to the internet, and self-identify as being able to speak and read English. We will exclude adults with substantial health conditions (e.g., congestive heart failure, diagnosed dementia); those anticipating further surgery (e.g. LLA revision) as identified by our study sites’ physiatrists; and an ability to use a tablet (e.g./ using hands for typing). Given these criteria, the results of this study will not be generalizable to all adults with LLA such as the very young and those who do not speak English. As most people with LLA are >50 years old and more than 90% of the population of BC and ON speak English, we are confident of obtaining a representative sample.

**2.6 Duration of Intervention:** Participants in the SMART group will receive the intervention for six weeks. Participants in the control group will be contacted weekly for a similar 6 week period.

**2.7** **Frequency and Duration of Follow-up:** There are six weekly SMART modules with each taking approximately 20 to 30 minutes to complete. Individuals will be encouraged to complete one module a week to reduce any perceived burden, and gradually integrate the information. Participants from both groups will receive a weekly contact: SMART group participants will receive a 20 minute telephone call/Zoom video call from peers, and the Control group participants one email or phone call by trainer.

**2.8 Outcome Measures:**

Note: all measures that involve participants walking will be conducted under remote supervision via Zoom.

***Primary Outcome:*** In contrast to medical interventions, many eHealth interventions have no pre-eminent outcome (e.g., reduced hypertension).^66^ Our primary outcome was selected to address the goals of the self-management program (patient perceived problems^22, 67^) identified in our previous work which is walking. We operationalized this as the Timed Up and Go Test (TUG), a performance measure of walking capacity -- deemed the strongest determinant of health-related quality of life and prosthetic walking proficiency in adults with LLA.^68^ It is widely used in intervention studies of the LLA population,^69^ with strong evidence of reliability, validity, and responsiveness.^37, 70^ The ability to walk longer distance permits people to explore and engage with their environment, which directly influences their choice of activities and social participation.^71^ Poor walking capacity may eventually lead to prosthetic abandonment.^72^ The Timed Up and Go (TUG) is a functional assessment that assesses aspects of mobility including the sit to stand transitions, gait initiation, acceleration and deceleration, and turning (Clemens et al., 2018). The TUG has been shown to be both a reliable and valid measure to evaluate how individuals with lower limb amputation function within their environment (Clemens et al., 2018). A mobile version of the TUG has also been shown to have excellent validity and reliability in the amputee population, making it an ideal tool for a mobile platform (Clemens et al., 2018). Participants will be asked to use a 48-cm-high armchair and mark a spot 3 meters from the front legs of the chair (Clemens et al., 2018). Participants would then be timed, starting from a sitting position, then standing and walking 3 meters, turning around and walking back and sitting back in the chair Clemens et al., 2018).

**Ambulatory Self-Confidence Questionnaire (ASCQ).** Perceived self-efficacy (confidence) includes the concept that one can exercise control over their health-related outcomes and activities and can therefore be an indicator in individuals perceived confidence in ambulation (Bandura, 2004). The ASCQ is a 22-item questionnaire where participants are asked to rate questions on a scale from 0 (not confident at all) to 10(extremely confident). (Asano et al., 2007). The ASCQ has been shown to have both reliability and validity in community-dwelling older adults (Asano et al., 2007).

**Timed Up and Go (TUG) Test.** The TUG assesses an individual’s ability to stand from a sitting position, walk three meters, turn around, walk back and sit back in the chair.^87^ It captures aspects of mobility capacity (what an individual can do)^86^ including sit-to-stand transitions, gait initiation, acceleration and deceleration, and turning.^87^

***Secondary outcomes*** are aligned with the performance objective of SMART and adults with LLA challenges (mentioned in section 1.2.) as well as patient-reported outcomes to capture patients’ views of their symptoms, and their functional status,^61, 73^ including:

- Physical Activity Identity (PAI) scale is a 9-item measure of an individual's identification with exercise as an integral part of the concept of self,^38^ with high test-retest reliability and good validity. We will use a modified version of the PAI questioning the extent that physical activity is considered a part of an individual’s aspect of self.^74^
- Activities Specific Balance Confidence Scale (ABC) is a 16-item self-reported measure of balance self-efficacy during ambulatory activities with total score of 0 to 100.^40^
- Euro Quality of Life – Five Level Instrument (EQ-5D-5L) assesses five dimensions of health-related quality of life: mobility, self-care, usual activities, pain/discomfort, and anxiety/depression.^88^
- Tenacious goal pursuit (TGP) assesses the tendency to persist and increase effort in pursuing goals facing obstacles.^89^
- Action planning scale (AP) assesses whether people had formed a plan which links goal-directed behavior to environmental cues by identifying when, where, and how to act.^90^ We will assess the AP for “exercising”, “skin monitoring”, and “cleaning the prosthesis”.
- Self-Management Assessment Scale (SMASc) assesses five domains that are important for an effective self-management, including “knowledge, goals for future, daily routines, emotional adjustment, and social support”.^91^
- *The Center for Epidemiologic Studies Depression Scale (CES-D)* is used to assess depressive symptoms over the past week in adults with LLL.^92-94^ Depression is an important health outcome in adults with LLL.
- *Revised Amputee Body Image Scale (ABIS-R)* assesses feelings about the body experienced after amputation.^95,96^ Poorer perceived body image is associated with depression and lower level of prosthetic satisfaction.^97^
- *Pain* is common in individuals with LLL and can adversely affect quality of life. We will assess the following: residual limb pain (pain in the remaining part of the amputated site); phantom limb pain (in missing part of the limb); and phantom sensation (in missing part of the limb). The intensity of pain and sensation in the past week will be assessed using a 10-cm visual analogue scale (VAS), ranging from 0 (which indicates no pain or sensation) to 10 (which indicates the worst pain or sensation possible).
- *Short Musculoskeletal Function Assessment (SMFA)* assesses individuals' perceived health status.^98,99^ The SMFA includes 46 items in three sections: difficulty with daily activities (items 1 to 25), experiencing problems because of injury (items 26 to 34), and the extent to which the person is bothered by the problems (items 35 to 46). In this study, we will use items 1 to 34, which cover the functional assessment.
- *Self-Report Habit Index (SRHI)* assesses the automaticity of a behavior and the extent to which individuals with LLL have integrated self-management tasks into their self-concept.^100,101^ We will assess the habit formation for “skin monitoring” and “cleaning the prosthesis,” which are important in managing a LLL.

***Implementation:*** Following the [RE-AIM](http://www.re-aim.org/) framework,^60^ five elements of implementation will be assessed including, reach (target population), effectiveness (effects of SMART), adoption (by setting/staff), implementation (dose delivered and received, fidelity to the intervention), and maintenance of the behavior (in target population and settings). During study, the research coordinator will keep the detailed logs to monitor fidelity to research protocols and review the online chart for the adherence to the SMART (dose). Moreover, ***participant experience*** will be identified with a brief 30-minute audio-recorded semi-structured in-person interview with men and women (n=20) from the experimental group (*Section 2.2*) by the researcher at the end of the study (T3) to explore the experience and acceptability of the SMART program, health related goals, and any perceived disadvantageous of SMART. Moreover, we will call any dropout participants to obtain their feedback.

***Sociodemographic and Clinical Characteristics***: We will collect descriptive characteristics at T1 (**Appendix E**) including, age, sex, gender, level of education, marital status, weight, height, and clinical variables, such as presence of comorbidities, level, cause, side, and date of amputation, discharge date, date in which prosthetic training has been started, and date in which patient received the permanent prosthesis. We will also collect the participants’ self-report encounters with outpatient rehab care providers (e.g., PT, OT) and surgical consultations throughout the six-week intervention.

A cognitive screen, the Montreal Cognitive Assessment (MoCA) will be used to screen for cognitive impairments.

**2.9 Measuring outcomes at follow-up:** We will collect data for intervention and control groups at three time points (**Appendix E**): T1=baseline; T2=within one week of completing SMART intervention; T3=retention period of four weeks post-intervention. The one-on-one semi-structured interviews with the SMART group to determine impressions of the protocol and intervention will be conducted at T3. All data collection will occur through online survey link on Qualtrics platform hosted by University of British Columbia. All sessions will be performed on Zoom video call.

**2.10 Sample Size:** Our sample size calculation is based on identifying SMART is more effective than standard care from a clinical point of view (Clinical Superiority design),^75^ and minimize any potential waste of resources.^76^ We are planning to compare the 2-MWT using a ratio 1:1. In a previous study, the mean (SD) of clinically important change in distance walked in 2 minutes at discharge from the rehab facility was 13.6 (19.9) m in adults with unilateral LLA (mean age = 66 y).^37^ Assuming minimum important difference of 13.6 m, we will need to enroll a minimum 34 participants per group to be enable to reject a null hypothesis of no difference in means of walking capacity between the 2 groups with 80% power. The type-I error probability associated with this 2-sided test of the null hypothesis is 0.05. Our sample size is based on the feasibility of recruiting 2 adults aged over 50 years with an LLA, per site within 18 months, and with 68 participants at final assessment (accounting for participant attrition) we can have enough power to detect a difference in the primary outcome, i.e., 2-MWT. Acknowledging the challenges with participants,^77^ 20% attrition rate, and non-compliance rate in RCTs, we will recruit 82 participants (41 per arm including both men and women).The target sample size will also facilitate estimates for adherence, and the number of participants with certain covariates (e.g., sex, gender), and inform the sample size calculation and stratification for the implementation study.

**2.11 Health services research issues:** Cost and cost-effectiveness of eHealth interventions have been demonstrated for patients with chronic disease conditions.^78^ However, we will not do a formal cost analysis in this study.

**2.12 Recruitment:** We anticipate it will take 3 years to complete this study **(Fig. 2)**. Eligible candidates will be identified from the Toronto, London, and Vancouver amputee clinics, as well as hospitals in 4 additional British Columbia Health Authorities: Fraser Health, Interior Health, Northern Health, and Vancouver Island Health. Invitation to participate will be provided through letters of information sent electronically or verbally by clinicians at each facility. Volunteers will contact the research coordinator or provide their verbal permission to their clinician to share their contact information (name and email/phone #) with the research coordinator. Volunteers will be screened by telephone upon contacting the research coordinator with an expression of interest to participate in the study. Upon successful screening/enrollment and baseline data collection (T1) the *research coordinator* will log onto the online randomization system to determine the next allocation within 48 hours, then, participant contact information will be forwarded to the appropriate *group Trainer* **(Appendix E)**. To mitigate recruitment challenges, we will offer an honorarium of $25 dollars per data collection session (T1-T3) to both groups. An additional $25 will be given to SMART group participants who participate in the T3 qualitative interview.

**2.13 Compliance:** The trainer of the SMART group will monitor the activity of participants through a website and will contact the participant to inquire about reasons for inactivity and troubleshoot any problems including technical issues. We also will explore the acceptability of SMART with the qualitative component of the study. Control group subjects will receive weekly contact.

**2.14 Loss to follow-up:** To mitigate loss to follow-up study coordinators will maintain contact with participants once every 4 weeks until study completion in addition to the trainer contact. Use of this strategy in a prior study by Imam and **NPA** **Miller**, et al^30^ demonstrated <20% loss to follow up.

**2.15 Study Centers:** We will recruit 82 individuals, equating to 20 or 21 participants per site. Multiple sites were chosen to understand the diversity of LLA population and health services: In British Columbia, we will recruit from GF Strong and Holy Family Hospital in Vancouver, BC, where approximately 100 adults with new LLA receive care each year. Participants will also be recruited from hospitals in 4 additional British Columbia Health Authorities: Fraser Health, Interior Health, Northern Health, and Vancouver Island Health; Sunnybrook and West Park in Toronto, each with over 100 inpatients per year; and Parkwood Hospital in London, ON, where more than 75 older adults with new LLA receive care. Our community partner Barber Prosthetics (Dave Moe and Brittany Pousett) will assist with recruitment through their extensive network in Metro Vancouver (Burnaby to Hope).


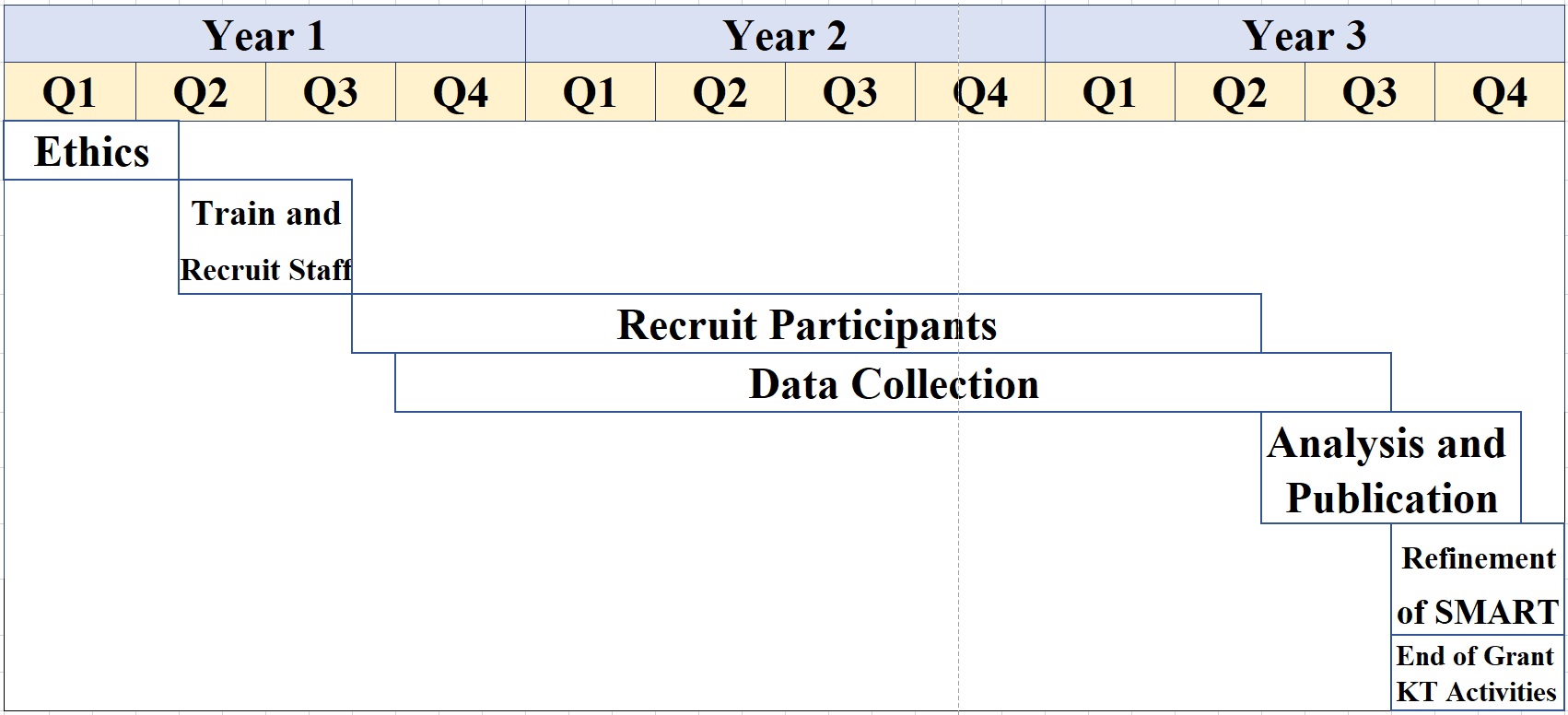


**Figure 2: Study Timeline**

**2.16 Data Analyses:** The assumptions for parametric analyses will be evaluated. Normal distribution will be evaluated using the one-sample Kolmogorov-Smirnov test. Qualitative comparison of variable balance will be made, including determination of prognostic importance. Descriptive statistics of training time and number of training sessions for the treatment group (collected via tablet usage data) will be used to evaluate dose-response and adherence.

***Clinical Outcomes:*** Analyses will be conducted on a per-protocol basis (i.e., participants who completed the study) and then on an intention-to-treat basis for comparison (i.e., using multiple imputation).^79^ All primary and secondary outcomes will be compared between the intervention and control groups, using analysis of covariance (ANCOVA), controlling for baseline score and inpatient and outpatient units. To explore the findings, ANCOVA will be used to compare secondary clinical outcomes between groups. Endpoint and change score analysis of variance (ANOVA) tests will also be conducted to compare precision with ANCOVA for sensitivity analyses. To glean a better understanding, we will disaggregate the data by sex/gender and *key outcomes* (e.g., walking capacity and physical activity identity) and explore differences in our study.

***Study and intervention fidelity, and participant experience:*** All audio-recordings of the qualitative interviews at T3 will be transcribed verbatim. Thematic data analyses^80^ will be used to identify key themes in the qualitative interviews. Three methods will be used to promote trustworthiness. Field notes will be used as a self-reflexive tool and member checking, (i.e., key participants will be invited to review the findings to determine how well they resonate with them). Multiple investigators will be involved in data coding as a form of triangulation. Interview results will complement the quantitative results to provide a more in-depth assessment of benefits and user acceptability.^59^

**2.17 Frequency of analyses:** An interim data analyses will be performed on the primary outcome when 50% of participants have completed T2. An independent blinded statistician will conduct the analyses and report findings to the Data and Safety Monitoring Committee (see *section 3.3*). If the results indicate the SMART or control arm is superior and sufficient information is available, we will terminate the trial to reduce subject burden.

**2.18 Sub-group analyses:** We will do a sub-group analysis to assess the differences in outcomes regards to sex, gender and amputation cause (Diabetes and dysvasuclar vs. others).

**References**:

1. Tossavainen PJ. Co-create with stakeholders: Action research approach in service development. Action Research. 2017;15(3):276-93.

2. Curran GM, Bauer M, Mittman B, Pyne JM, Stetler C. Effectiveness-implementation hybrid designs: combining elements of clinical effectiveness and implementation research to enhance public health impact. Medical care. 2012;50(3):217.

3. Imam B, Miller WC, Finlayson HC, Eng JJ, Jarus T. Incidence of lower limb amputation in Canada. Canadian Journal of Public Health. 2017;108(4):7.

4. Kayssi A, de Mestral C, Forbes TL, Roche-Nagle G. Predictors of hospital readmissions after lower extremity amputations in Canada. Journal of Vascular Surgery. 2016;63(3):688-95.

5. Dreyer RP, van Zitteren M, Beltrame JF, Fitridge R, Denollet J, Vriens PW, et al. Gender differences in health status and adverse outcomes among patients with peripheral arterial disease. Journal of the American Heart Association. 2014;4(1):e000863-e.

6. Peek ME. Gender differences in diabetes-related lower extremity amputations. Clinical Orthopaedics and Related Research. 2011;469(7):1951-5.

7. Miller WC, Speechley M, Deathe AB. The prevalence and risk factors of falling and fear of falling among lower extremity amputees. Archives of Physical Medicine and Rehabilitation. 2001;82(8):1031-7.

8. Hagberg K, Brånemark R. Consequences of non-vascular trans-femoral amputation: a survey of quality of life, prosthetic use and problems. Prosthetics and Orthotics International. 2001;25(3):186-94.

9. Hamill R, Carson S, Dorahy M. Experiences of psychosocial adjustment within 18 months of amputation: an interpretative phenomenological analysis. Disability and Rehabilitation. 2010;32(9):729-40.

10. Rybarczyk B, Edwards R, Behel J. Diversity in adjustment to a leg amputation: case illustrations of common themes. Disability and Rehabilitation. 2004;26(14-15):944-53.

11. Miller WC, Deathe AB. The influence of balance confidence on social activity after discharge from prosthetic rehabilitation for first lower limb amputation. Prosthetics and Orthotics International. 2011;35(4):379-85.

12. Miller WC, Deathe AB, Speechley M, Koval J. The influence of falling, fear of falling, and balance confidence on prosthetic mobility and social activity among individuals with a lower extremity amputation. Archives of Physical Medicine and Rehabilitation. 2001;82(9):1238-44.

13. Horgan O, MacLachlan M. Psychosocial adjustment to lower-limb amputation: a review. Disability and Rehabilitation. 2004;26(14-15):837-50.

14. MacKenzie EJ, Castillo RC, Jones AS, Bosse MJ, Kellam JF, Pollak AN, et al. Health-care costs associated with amputation or reconstruction of a limb-threatening injury. The Journal of Bone and Joint Surgery. 2007;89(8):1685-92.

15. Quality AfHRa. HCUP Nationwide Inpatient Sample (NIS): Healthcare Cost and Utilization Project (HCUP). Rockville, MD2009.

16. Franklin H, Rajan M, Tseng C-L, Pogach L, Sinha A, Mph M. Cost of lower-limb amputation in US veterans with diabetes using health services data in fiscal years 2004 and 2010. J Rehabil Res Dev. 2014;51(8):1325-30.

17. Graz H, D'Souza VK, Alderson DEC, Graz M. Diabetes-related amputations create considerable public health burden in the UK. Diabetes Res Clin Pract. 2018;135:158-65.

18. Imam B, Miller WC, Finlayson H, Eng J, Jaurs T. Provision of inpatient rehabilitation to individuals with lower limb amputation in Canada. Physiotherapy Canada. 2019 (Submitted).

19. Czerniecki JM, Turner AP, Williams RM, Hakimi KN, Norvell DC. The effect of rehabilitation in a comprehensive inpatient rehabilitation unit on mobility outcome after dysvascular lower extremity amputation. Archives of Physical Medicine and Rehabilitation. 2012;93(8):1384-91.

20. Imam B. Incidence and rehabilitation of lower limb amputation in Canada, and feasibility of a novel training program. Vancouver: The University of British Columbia; 2017.

21. Dillingham TR, Pezzin LE, MacKenzie EJ. Discharge destination after dysvascular lower-limb amputations. Archives of Physical Medicine and Rehabilitation. 2003;84(11):1662-8.

22. Lorig KR, Holman HR. Self-management education: history, definition, outcomes, and mechanisms. Annals of Behavioral Medicine. 2003;26(1):1-7.

23. Schulman-Green D, Jaser S, Martin F, Alonzo A, Grey M, McCorkle R, et al. Processes of self-management in chronic illness. Journal of Nursing Scholarship. 2012;44(2):136-44.

24. World Health Organization. Global observatory for eHealth series - Volume 3.2011.

25. Cotterez A, Durant N, Agne A, Cherrington A. Internet interventions to support lifestyle modification for diabetes management: A systematic review of the evidence. Journal of Diabetes and Its Complications. 2014;28(2):243-51.

26. Payne HE, Lister C, West JH, Bernhardt JM. Behavioral functionality of mobile apps in health interventions: a systematic review of the literature. JMIR mHealth and uHealth. 2015;3(1).

27. van Twillert S, Postema K, Geertzen JH, Lettinga AT. Incorporating self-management in prosthetic rehabilitation: case report of an integrated knowledge-to-action process. Physical Therapy. 2015;95(4):640-7.

28. Christiansen CL, Miller MJ, Murray AM, Stephenson RO, Stevens-Lapsley JE, Hiatt WR, et al. Behavior-change intervention targeting physical function, walking, and disability after dysvascular amputation: A randomized controlled pilot trial. Arch Phys Med Rehabil. 2018.

29. Godlwana L, Stewart A, Musenge E. The effect of a home exercise intervention on persons with lower limb amputations: a randomized controlled trial. Clin Rehabil. 2019:0269215519880295.

30. Imam B, Miller WC, Finlayson H, Eng JJ, Jarus T. A randomized controlled trial to evaluate the feasibility of the Wii Fit for improving walking in older adults with lower limb amputation. Clinical Rehabilitation. 2017;31(1):82-92.

31. Littman AJ, Haselkorn JK, Arterburn DE, Boyko EJ. Pilot randomized trial of a telephone-delivered physical activity and weight management intervention for individuals with lower extremity amputation. Disability and health journal. 2018.

32. Rothgangel A, Braun S, Winkens B, Beurskens A, Smeets R. Traditional and augmented reality mirror therapy for patients with chronic phantom limb pain (PACT study): results of a three-group, multicentre single-blind randomized controlled trial. Clin Rehabil. 2018;32(12):1591-608.

33. Wegener ST, Mackenzie EJ, Ephraim P, Ehde D, Williams R. Self-management improves outcomes in persons with limb loss. Archives of Physical Medicine and Rehabilitation. 2009;90(3):373-80.

34. Esfandiari E, Miller CA, King S, Ashe MC, Mortenson BW, editors. Education After Lower Limb Amputation: A Qualitative Study on Clients’ Perspectives to Develop an Online Self-Management Program. RehabWeek; 2019; Toronto, Canada.

35. Esfandiari E, Miller WC, King S, Ashe MC, Mortenson BW, editors. Education After Amputation: A Qualitative Study on Individuals’ with Lower Limb Amputation Perspectives to Develop an Online Self-Management Program. BC Physio Education Forum 2020; 2020; Vancouver, BC, Canada.

36. Esfandiari E, Miller WC, King S, Ashe MC, W Mortenson B. Education After Lower Limb Amputation: A Qualitative Study on Clinicians’ Perspectives. The Ontario Association for Amputee Care Conference; Toronto, Ontario2019.

37. Brooks D, Parsons J, Hunter JP, Devlin M, Walker J. The 2-minute walk test as a measure of functional improvement in persons with lower limb amputation. Archives of Physical Medicine and Rehabilitation. 2001;82(10):1478-83.

38. Anderson DF, Cychosz CM. Development of An Exercise Identity Scale. Percept Mot Skills. 1994;78(3):747-51.

40. Miller WC, Deathe AB, Speechley M. Psychometric properties of the Activities-specific Balance Confidence Scale among individuals with a lower-limb amputation. Archives of Physical Medicine and Rehabilitation. 2003;84(5):656-61.

41. Esfandiari E, Miller WC, Berardi A, Mortenson WB, Ashe MC. Behavior change techniques used in telehealth interventions for mobility after lower limb musculoskeletal surgery: A systematic review and meta-analysis (Submitted). J Bone Joint Surg. 2020.

42. Michie S, Ashford S, Sniehotta FF, Dombrowski SU, Bishop A, French DP. A refined taxonomy of behaviour change techniques to help people change their physical activity and healthy eating behaviours: The CALO-RE taxonomy. Psychology and Health. 2011;26(11):1479-98.

43. Michie S, Richardson M, Johnston M, Abraham C, Francis J, Hardeman W, et al. The Behavior Change Technique Taxonomy (v1) of 93 hierarchically clustered techniques: Building an international consensus for the reporting of behavior change interventions. Ann Behav Med. 2013;46(1):81-95.

44. Bandura A. Self-efficacy: toward a unifying theory of behavioral change. Psychological Review. 1977;84(2):191.

45. Schwarzer R, Lippke S, Luszczynska A. Mechanisms of health behavior change in persons with chronic illness or disability: the Health Action Process Approach (HAPA). Rehabilitation Psychology. 2011;56(3):161.

46. Cane J, O’Connor D, Michie S. Validation of the theoretical domains framework for use in behaviour change and implementation research. Implement Sci. 2012;7(1):37.

47. Michie S, Johnston M, Abraham C, Lawton R, Parker D, Walker A. Making psychological theory useful for implementing evidence based practice: a consensus approach. BMJ Qual Saf. 2005;14(1):26-33.

48. Tremblay M, Amber S, Esfandiari E, King S, Miller WC, editors. Feasibility and acceptability of Self-Management for Amputee Rehabilitation using Technology. Canadian Association of Occupational Therapists; 2020; Saskatoon, Canada.

49. Lorig KR, Sobel DS, Stewart AL, Brown BW, Bandura A, Ritter P, et al. Evidence suggesting that a chronic disease self-management program can improve health status while reducing hospitalization: A randomized trial. Medical Care. 1999;37(1):5-14.

50. Dicianno BE, Parmanto B, Fairman AD, Crytzer TM, Yu DX, Pramana G, et al. Perspectives on the evolution of mobile (mHealth) technologies and application to rehabilitation. Phys Ther. 2015;95(3):397-405.

51. Jayakumar N, Brunckhorst O, Dasgupta P, Khan MS, Ahmed K. e-Learning in surgical education: A systematic review. J Surg Educ. 2015;72(6):1145-57.

52. Gruner D, Pottie K, Archibald D, Allison J, Sabourin V, Belcaid I, et al. Introducing global health into the undergraduate medical school curriculum using an e-learning program: a mixed method pilot study. BMC Med Educ. 2015;15(1):142.

53. Lorig KR, Ritter P, Stewart AL, Sobel DS, Brown BW, Bandura A, et al. Chronic Disease Self-Management Program: 2-Year Health Status and Health Care Utilization Outcomes. Medical Care. 2001;39(11):1217-23.

54. Uustal H. Prosthetic rehabilitation issues in the diabetic and dysvascular amputee. Physical Medicine and Rehabilitation Clinics of North America. 2009;20(4):689-703.

55. Table 358-0154-Canadian Internet use survey, Internet use, location of use, household income and age group for Canada and regions, occasional (percent) [Internet]. Statistics Canada. 2012.

56. Stineman MG, Kwong PL, Xie D, Kurichi JE, Ripley DC, Brooks DM, et al. Prognostic differences for functional recovery after major lower limb amputation: effects of the timing and type of inpatient rehabilitation services in the Veterans Health Administration. The American Academy of Physical Medicine and Rehabilitation Journal 2010;2(4):232-43.

57. Gailey R, McFarland LV, Cooper RA, Czerniecki J, Gambel JM, Hubbard S, et al. Unilateral lower-limb loss: Prosthetic device use and functional outcomes in servicemembers from Vietnam war and OIF/OEF conflicts. J Rehabil Res Dev. 2010;47(4):317-32.

58. Street H, O'Connor M, Robinson H. Depression in older adults: Exploring the relationship between goal setting and physical health. International Journal of Geriatric Psychiatry: A journal of the psychiatry of late life and allied sciences. 2007;22(11):1115-9.

59. Mortenson BW, Oliffe JL. Mixed methods research in occupational therapy: A survey and critique. OTJR: Occupation, Participation, Health. 2009;29(1):14-23.

60. Glasgow RE, Vogt TM, Boles SM. Evaluating the public health impact of health promotion interventions: the RE-AIM framework. Am J Public Health. 1999;89.

61. Black N. Patient reported outcome measures could help transform healthcare. Bmj. 2013;346:f167.

62. Hoffmann TC, Glasziou PP, Boutron I, Milne R, Perera R, Moher D, et al. Better reporting of interventions: template for intervention description and replication (TIDieR) checklist and guide. BMJ. 2014;348:g1687.

63. Gutnick D, Reims K, Davis C, Gainforth H, Jay M, Cole S. Brief action planning to facilitate behavior change and support patient self-management. Journal of Clinical Outcomes Management. 2014;21(1):17-29.

64. Lundahl B, Moleni T, Burke BL, Butters R, Tollefson D, Butler C, et al. Motivational interviewing in medical care settings: a systematic review and meta-analysis of randomized controlled trials. Patient education and counseling. 2013;93(2):157-68.

65. Goldman ML, Ghorob A, Eyre SL, Bodenheimer T. How Do Peer Coaches Improve Diabetes Care for Low-Income Patients?:A Qualitative Analysis. The Diabetes Educator. 2013;39(6):800-10.

66. Baker TB, Gustafson DH, Shaw B, Hawkins R, Pingree S, Roberts L, et al. Relevance of CONSORT reporting criteria for research on eHealth interventions. Patient Education and Counseling. 2010;81:S77-S86.

67. Murray E. Web-based interventions for behavior change and self-management: Potential, pitfalls, and progress. Medicine 20. 2012;1(2):e3.

68. van der Schans CP, Geertzen JH, Schoppen T, Dijkstra PU. Phantom pain and health-related quality of life in lower limb amputees. Journal of Pain and Symptom Management. 2002;24(4):429-36.

69. Deathe AB, Wolfe DL, Devlin M, Hebert JS, Miller WC, Pallaveshi L. Selection of outcome measures in lower extremity amputation rehabilitation: ICF activities. Disability and Rehabilitation. 2009;31(18):1455-73.

70. Pin TW. Psychometric properties of 2-Minute Walk Test: A systematic review. Archives of Physical Medicine and Rehabilitation. 2014;95(9):1759-75.

71. Munin MC, Espejo-De Guzman MC, Boninger ML, Fitzgerald SG, Penrod LE, Singh J. Predictive factors for successful early prosthetic ambulation among lower-limb amputees. Journal of Rehabilitation Research and Development. 2001;38(4):379-84.

72. Geertzen JH, Bosmans JC, Van Der Schans CP, Dijkstra PU. Claimed walking distance of lower limb amputees. Disability and Rehabilitation. 2005;27(3):101-4.

73. Willke RJ, Burke LB, Erickson P. Measuring treatment impact: a review of patient-reported outcomes and other efficacy endpoints in approved product labels. Control Clin Trials. 2004;25(6):535-52.

74. Strachan SM, Brawley LR, Spink K, Glazebrook K. Older adults' physically-active identity: Relationships between social cognitions, physical activity and satisfaction with life. Psychology of Sport and Exercise. 2010;11(2):114-21.

75. Zhong B. How to calculate sample size in randomized controlled trial? J Thorac Dis. 2009;1(1):51.

76. Farrokhyar F, Reddy D, Poolman RW, Bhandari M. Why perform a priori sample size calculation? Canadian journal of surgery Journal canadien de chirurgie. 2013;56(3):207-13.

77. Teare MD, Dimairo M, Shephard N, Hayman A, Whitehead A, Walters SJ. Sample size requirements to estimate key design parameters from external pilot randomised controlled trials: a simulation study. Trials. 2014;15(1):264.

78. Udsen FW, Hejlesen O, Ehlers LH. A systematic review of the cost and cost-effectiveness of telehealth for patients suffering from chronic obstructive pulmonary disease. Journal of Telemedicine and Telecare. 2014;20(4):212-20.

79. Armijo-Olivo S, Warren S, Magee D. Intention to treat analysis, compliance, drop-outs and how to deal with missing data in clinical research: a review. Physical Therapy Reviews. 2009;14(1):36-49.

80. Braun V, Clarke V. Using thematic analysis in psychology. Qualitative Research in Psychology. 2006;3(2):77-101.

81. Cooke L. Assessing concurrent Think-Aloud protocol as a usability test method: A technical communication approach. IEEE Transactions on Professional Communication. 2010;53(3):202-15.

82. Senra H, Oliveira RA, Leal I, Vieira C. Beyond the body image: a qualitative study on how adults experience lower limb amputation. Clinical Rehabilitation. 2012;26(2):180-91.

83. Brooks D, Hunter JP, Parsons J, Livsey E, Quirt J, Devlin M. Reliability of the two-minute walk test in individuals with transtibial amputation. Archives of Physical Medicine and Rehabilitation. 2002;83(11):1562-5.

84. Pohjolainen T, Alaranta H. Predictive factors of functional ability after lower-limb amputation. Ann Chir Gynaecol. 1991;80(1):36-9.

85. Craig P, Dieppe P, Macintyre S, Michie S, Nazareth I, Petticrew M. Developing and evaluating complex interventions: The new Medical Research Council guidance. International Journal of Nursing Studies. 2013;50(5):587-92.

86. Clemens SM, Gailey RS, Bennett CL, et al. The Component Timed-Up-and-Go test: the utility and psychometric properties of using a mobile application to determine prosthetic mobility in people with lower limb amputations. Clin Rehabil 2018; 32: 388-397. 2017/09/02. DOI: 10.1177/0269215517728324.

87. Deathe AB, Wolfe DL, Devlin M, et al. Selection of outcome measures in lower extremity amputation rehabilitation: ICF activities. Disability and Rehabilitation 2009; 31: 1455-1473. DOI: 10.1080/09638280802639491.

88 Herdman M, Gudex C, Lloyd A, Janssen M, Kind P, Parkin D, et al. Development and preliminary testing of the new five-level version of EQ-5D (EQ-5D-5L). Quality of life research. 2011;20(10):1727-36.

89 Brandtstädter J, Renner G. Tenacious goal pursuit and flexible goal adjustment: explication and age-related analysis of assimilative and accommodative strategies of coping. Psychol Aging. 1990;5(1):58.

90. Sniehotta FF, Schwarzer R, Scholz U, Schüz B. Action planning and coping planning for long‐term lifestyle change: theory and assessment. European Journal of Social Psychology. 2005;35(4):565-76.

91. Öberg U, Hörnsten Å, Isaksson U. The Self-Management Assessment Scale: Development and psychometric testing of a screening instrument for person-centred guidance and self-management support. Nursing Open. 2019;6(2):504-13. doi: <https://doi.org/10.1002/nop2.233>.

92. Dunn DS. Well-being following amputation: Salutary effects of positive meaning, optimism, and control. Rehabilitation Psychology. 1996;41(4):285.

93. Hebert JS, Wolfe DL, Miller WC, Deathe AB, Devlin M, Pallaveshi L. Outcome measures in amputation rehabilitation: ICF body functions. Disability and Rehabilitation. 2009;31(19):1541-54. doi: 10.1080/09638280802639467.

94. Hanley MA, Jensen MP, Ehde DM, Hoffman AJ, Patterson DR, Robinson LR. Psychosocial predictors of long-term adjustment to lower-limb amputation and phantom limb pain. Disability and Rehabilitation. 2004;26(14-15):882-93.

95. Hebert JS, Wolfe DL, Miller WC, Deathe AB, Devlin M, Pallaveshi L. Outcome measures in amputation rehabilitation: ICF body functions. Disability and Rehabilitation. 2009;31(19):1541-54. doi: 10.1080/09638280802639467.

96. Gallagher P, Horgan O, Franchignoni F, Giordano A, MacLachlan M. Body image in people with lower-limb amputation: A rasch analysis of the amputee body image scale. American Journal of Physical Medicine & Rehabilitation. 2007;86(3):205-15. doi: 10.1097/PHM.0b013e3180321439. PubMed PMID: 00002060-200703000-00008.

97. Breakey JW. Body image: the lower-limb amputee. JPO: Journal of Prosthetics and Orthotics. 1997;9(2):58-66.

98. Swiontkowski MF, Engelberg R, Martin DP, Agel J. Short Musculoskeletal Function Assessment Questionnaire: Validity, Reliability, and Responsiveness. Journal of bone and joint surgery American volume. 1999;81(9):1245-60. doi: 10.2106/00004623-199909000-00006.

99. Swiontkowski MF, Engelberg R, Martin DP, Agel J. Short musculoskeletal function assessment questionnaire: validity, reliability, and responsiveness. Orthopedic Trauma Directions. 2005;3(02):29-34.

100. Verplanken B, Orbell S. Reflections on past behavior: A self‐report index of habit strength. Journal of Applied Social Psychology. 2003;33(6):1313-30.

101. Lally P, Gardner B. Promoting habit formation. Health Psychology Review. 2013;7(sup1):S137-S58.

**Appendix A.** Overview of SMART content and Behavior Change Techniques


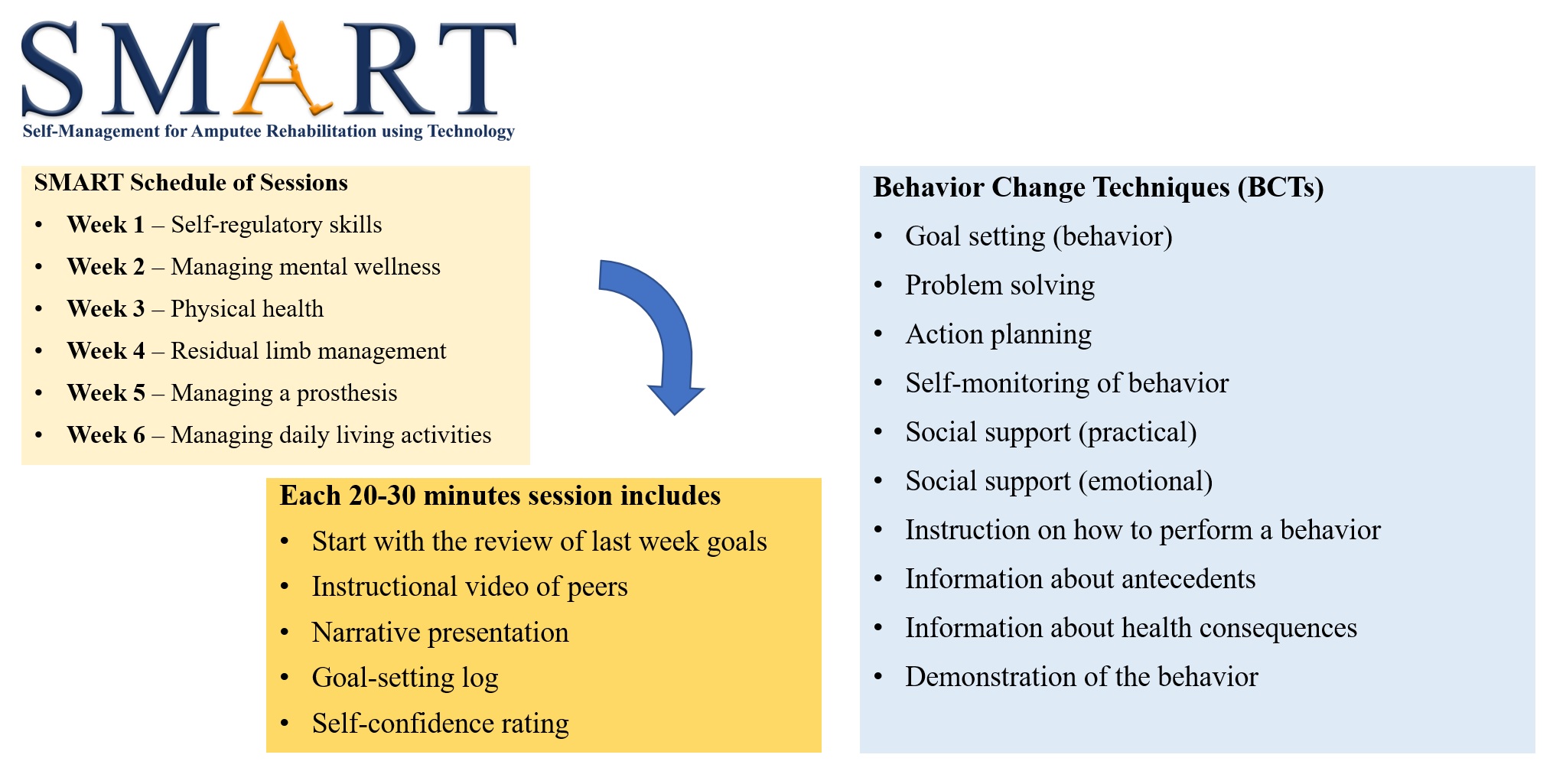


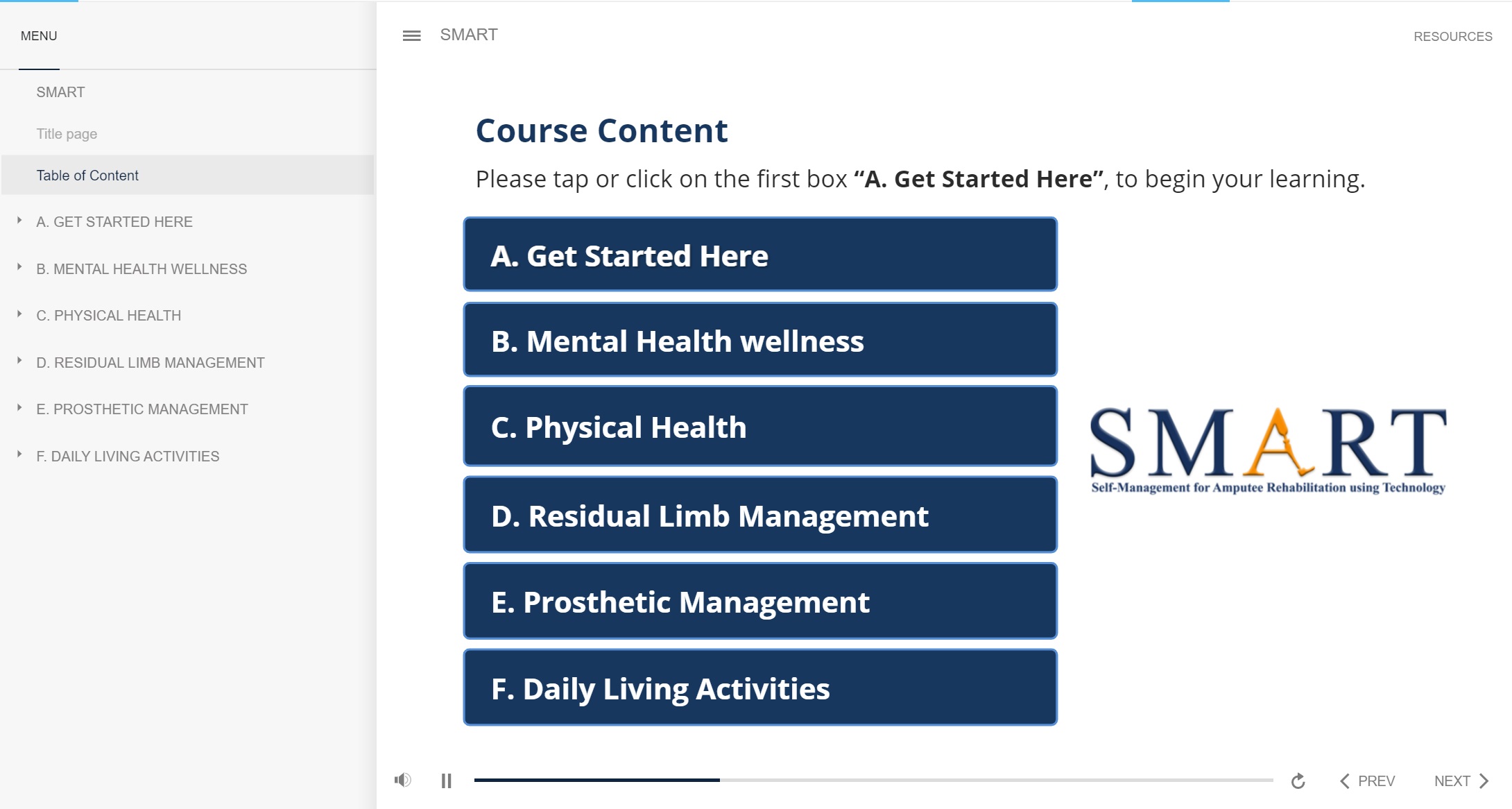


**Appendix B.** Template for Intervention Description and Replication (TIDieR) checklist for SMART

| **Name** | **Self-Management for Amputee Rehabilitation using Technology (SMART)** |
| --- | --- |
| Why (rationale and theory) | Following Lower Limb Amputation (LLA) there are so many challenges for the patient and their healthcare provider. These challenges are further complicated with depression, distorted body image, lack of coping strategies,^82^ and insufficient educational resources and training.^27, 33^ Additionally, the COVID-19 pandemic has impacted the delivery and accessibility of healthcare services.  To address these challenges, using a participatory action research  approach based on theoretical domains framework (TDF)^47^ we have designed SMART as an online educational and training platform for individuals with LLA to help them to manage their health-related conditions. |
| What (materials) | Participants in SMART group will have access to the SMART platform for 10 weeks. SMART includes 6 modules, about 20 to 30 minutes in length, about the self-regulatory skills, managing mental wellness, physical health, residual limb management, managing a prosthesis, and managing daily living activities. Participants will receive a weekly 20-minute telephone contact from peers for 6 weeks to facilitate motivation using goal setting and action planning. There is also a *resource icon* that provides more information and resources, such as timeline after LLA, and funding for prostheses. |
| What (Procedures) | It is a multi-site parallel RCT at BC (Vancouver Costal Health, Fraser Health, Interior Health, Northern Health, and Vancouver Island Health), Toronto, ON (West Park Healthcare Centre & Sunnybrook Health Sciences Centre), and London, ON (Parkwood Hospital). The research coordinator will provide the contact information for each participant, to his or her trainer. The subjects in SMART group will receive online training on using the online SMART platform. An additional icon in SMART enables direct *private email contact to the trainer,* so that participant questions or problems are quickly addressed. The trainer will be a registered physiotherapist (PT) or occupational therapist (OT) with more than 5 years clinical experience who will arrange the first telephone call of peers. If there is also no online activity within one week through SMART by subjects, the trainer will call the subject to foster motivation and troubleshoot the potential technical problems. |
| Who provided | The trainer will spend less than 2 hours to provide instruction for a participant to learn how to use the SMART, at baseline (T1). The SMART platform will be asynchronously monitored through a web portal by the trainer, who will observe participant progress and provide feedback if required. If there is no online activity within a 7-day (consecutive) period, the trainer will contact the participant to inquire the reasons for inactivity and troubleshoot any problems including technical issues.  Two peers will be identified by our clinician partners at each study site. Peers are mentors with LLA who support the individuals with LLA.^65^ The assessor will be a trained research assistant who will be blinded to participants assigned groups. The assessor will evaluate all participants at baseline (T1), end of the intervention (6-weeks) (T2), and 4-week retention period (T3). |
| How (modes of delivery) | SMART is an online, educational and training platform for individuals with LLA that is asynchronously monitored by a trainer through a website. SMART will be delivered via computer or tablet with support of peers. |
| Where | Participants will use one module of SMART per week. All participants will be evaluated by an assessor online via Zoom (a secure, UBC-based platform) at T1, T2, and T3. |

**Appendix C:** Behavioural change techniques used for contents of modules in Self-Management for Amputee Rehabilitation Using Technology (SMART)

| **BCTs** | **Self-regulatory skills** | **Mental wellness** | **Physical health** | **Residual limb management** | **Managing a prosthesis** | **Managing daily living activities** |
| --- | --- | --- | --- | --- | --- | --- |
| Goal setting (behaviour) | * | * | * | * | * | * |
| Problem solving |  | * | * | * | * | * |
| Action planning |  | * | * | * | * | * |
| Self-monitoring of behaviour |  |  | * | * | * | * |
| Social support (practical) | * | * | * | * | * | * |
| Social support (emotional) |  | * | * | * | * | * |
| Instruction on how to perform a behaviour | * | * | * | * | * | * |
| Information about antecedents |  | * | * | * | * | * |
| Information about health consequences |  | * | * | * |  |  |
| Demonstration of the behaviour |  |  | * | * | * | * |

**Appendix D.** Brief Action Planning (BAP) Flowchart

Developed by Steven Cole, Damara Gutnick, Connie Davis, Kathy Reims

Accessed from: <https://centrecmi.ca/wp-content/uploads/2018/11/BAP_flow_Chart_2016-08-08.pdf>


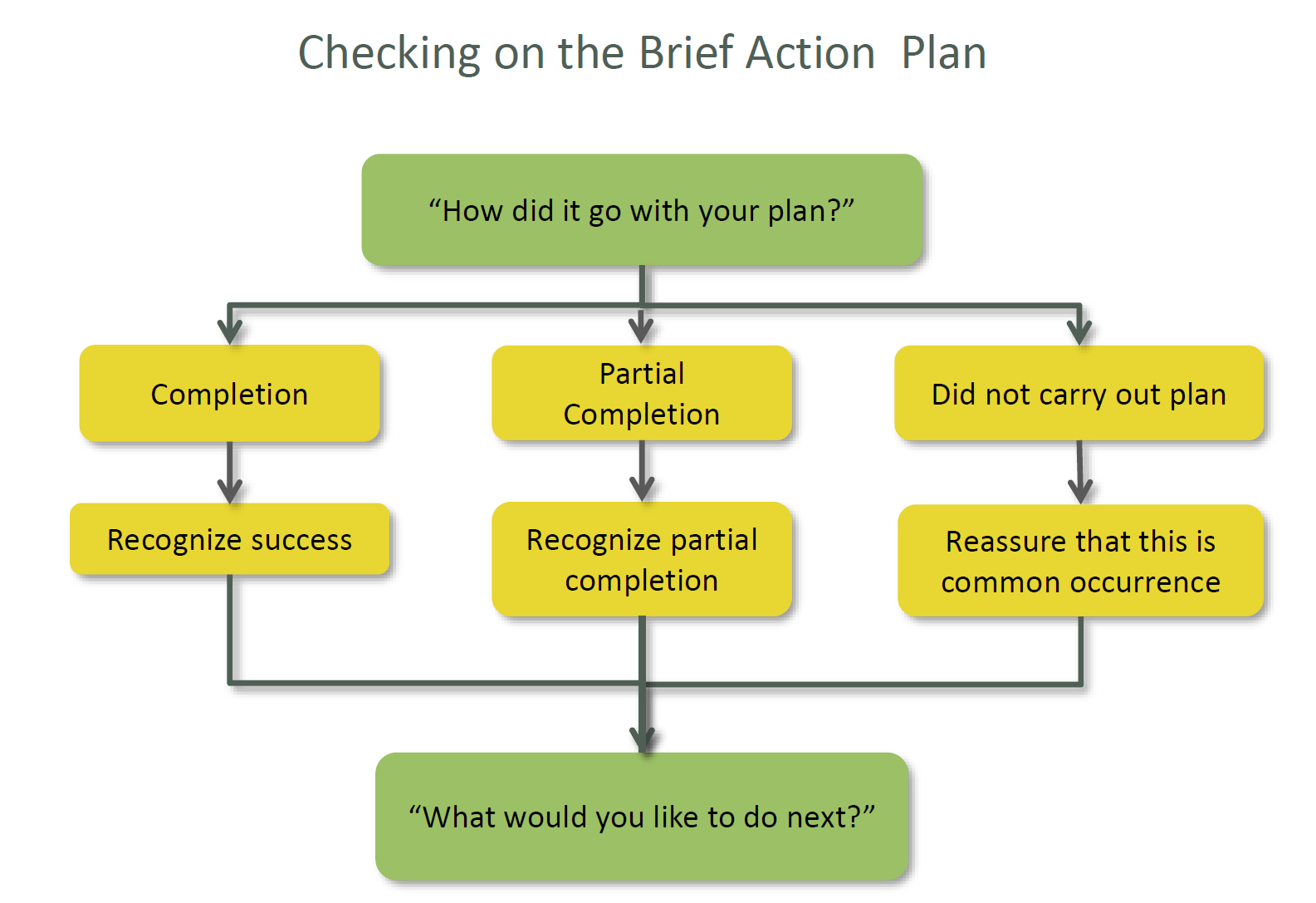


**Appendix E.** Study flowchart


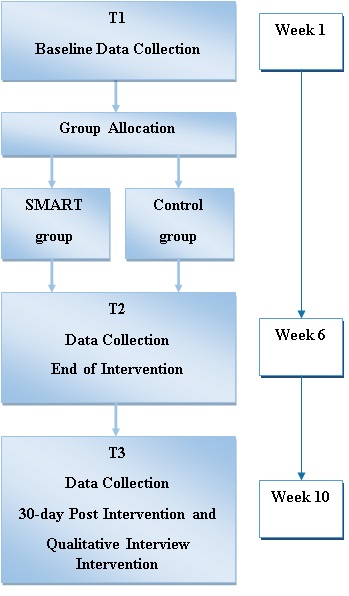

Supplement: S1 File — (DOC) [file pone.0278418.s002.doc]
